# Development and Technical Validation of an Immunoassay for the Detection of APP_669–711_ (Aβ_−3–40_) in Biological Samples

**DOI:** 10.3390/ijms21186564

**Published:** 2020-09-08

**Authors:** Hans W. Klafki, Petra Rieper, Anja Matzen, Silvia Zampar, Oliver Wirths, Jonathan Vogelgsang, Dirk Osterloh, Lara Rohdenburg, Timo J. Oberstein, Olaf Jahn, Isaak Beyer, Ingolf Lachmann, Hans-Joachim Knölker, Jens Wiltfang

**Affiliations:** 1 Department of Psychiatry and Psychotherapy, University Medical Center (UMG), Georg-August-University, D37075 Göttingen, Germany; petra.rieper@med.uni-goettingen.de (P.R.); silvia.zampar@med.uni-goettingen.de (S.Z.); oliver.wirths@medizin.uni-goettingen.de (O.W.); jonathan.vogelgsang@med.uni-goettingen.de (J.V.); lara.rohdenburg@stud.uni-goettingen.de (L.R.); Jens.Wiltfang@med.uni-goettingen.de (J.W.); 2 IBL International GmbH, Tecan Group Company, D-22335 Hamburg, Germany; Anja.Matzen@tecan.com; 3 Roboscreen GmbH, D-04129 Leipzig, Germany; dirk.osterloh@roboscreen.com (D.O.); ingolf.lachmann@roboscreen.com (I.L.); 4 Department of Psychiatry and Psychotherapy, Friedrich-Alexander-University of Erlangen-Nuremberg, D-91054 Erlangen, Germany; Timo.Oberstein@uk-erlangen.de; 5 Max-Planck-Institute of Experimental Medicine, Proteomics Group, D-37075 Göttingen, Germany; jahn@em.mpg.de; 6 Faculty of Chemistry, Technische Universität Dresden, D-01069 Dresden, Germany; isaak.beyer@web.de (I.B.); hans-joachim.knoelker@tu-dresden.de (H.-J.K.); 7 German Center for Neurodegenerative Diseases (DZNE), D-37075 Göttingen, Germany; 8 Neurosciences and Signaling Group, Institute of Biomedicine (iBiMED), Department of Medical Sciences, University of Aveiro, 3810-193 Aveiro, Portugal

**Keywords:** Alzheimer’s disease, biomarker, amyloid β, assay development, assay validation

## Abstract

The ratio of amyloid precursor protein (APP)_669–711_ (Aβ_−3–40_)/Aβ_1–42_ in blood plasma was reported to represent a novel Alzheimer’s disease biomarker. Here, we describe the characterization of two antibodies against the N-terminus of Aβ_−3–x_ and the development and “fit-for-purpose” technical validation of a sandwich immunoassay for the measurement of Aβ_−3–40_. Antibody selectivity was assessed by capillary isoelectric focusing immunoassay, Western blot analysis, and immunohistochemistry. The analytical validation addressed assay range, repeatability, specificity, between-run variability, impact of pre-analytical sample handling procedures, assay interference, and analytical spike recoveries. Blood plasma was analyzed after Aβ immunoprecipitation by a two-step immunoassay procedure. Both monoclonal antibodies detected Aβ_−3–40_ with no appreciable cross reactivity with Aβ_1–40_ or N-terminally truncated Aβ variants. However, the amyloid precursor protein was also recognized. The immunoassay showed high selectivity for Aβ_−3–40_ with a quantitative assay range of 22 pg/mL–7.5 ng/mL. Acceptable intermediate imprecision of the complete two-step immunoassay was reached after normalization. In a small clinical sample, the measured Aβ_42_/Aβ_−3–40_ and Aβ_42_/Aβ_40_ ratios were lower in patients with dementia of the Alzheimer’s type than in other dementias. In summary, the methodological groundwork for further optimization and future studies addressing the Aβ_42_/Aβ_−3–40_ ratio as a novel biomarker candidate for Alzheimer’s disease has been set.

## 1. Introduction

Alzheimer’s disease (AD) is a progressive neurodegenerative disorder that is responsible for the majority of dementia cases [1]. Amyloid plaques containing aggregated forms of amyloid β (Aβ) peptides represent one of the classical neuropathological hallmarks in AD brains [2,3]. Aβ is generated from the amyloid precursor protein (APP) by consecutive proteolytic cleavages executed by β- and γ-secretases [4]. Oligomeric aggregated forms of Aβ can impair synaptic function and have neurotoxic properties, and are thus believed to play a critical role in AD pathogenesis (for a recent review, see, for example, [5]). In recent years, a variety of N- and C-terminally truncated or modified versions of the canonical Aβ_1–40_ and Aβ_1–42_ species have been detected in amyloid plaques [6,7,8], and it appears that the exact length and amino sequence of both N- and C-termini affect aggregation tendency and neurotoxicity of the different Aβ-species [9,10]. For example, Aβ peptides starting with pyroglutamate at position 3 of the Aβ sequence (Aβ_N3pE–X_) were shown to be abundant in senile amyloid plaques [11]. This posttranslational modification seems to increase the oligomerization, fibrillization, and cytotoxicity of Aβ [12,13,14,15]. Interestingly, a recent solid-state NMR spectroscopy study suggested that the secondary structure of Aβ oligomers was only marginally affected by pyroglutamate-modification of the Aβ N-terminus [16]. Currently, no disease modifying treatments for AD targeting the underlying pathophysiological processes are available. Biochemical and imaging biomarkers of the pathological changes in AD are important for an early and differential dementia diagnosis, in order to identify subjects at high risk in preclinical stages and for participant selection in the context of clinical trials [17,18]. Recently, the ratios of the concentrations of specific variants of amyloid β (Aβ) in human blood plasma were discovered to predict brain amyloid pathology with high accuracy or to differentiate Alzheimer’s dementia patients and subjects with dementia due to other reasons. These plasma Aβ ratios include Aβ_42_/Aβ_40_ [19,20,21], Aβ_1–40_/Aβ_1–42_, and APP_669–711_/Aβ_1–42_ [22,23]. APP_669–711_ is a proteolytic fragment of the amyloid precursor protein (APP) that was identified in human blood plasma by immunoprecipitation followed by mass spectrometry [24]. The APP_669–711_ peptide starts three amino acids N-terminal to the canonical Aβ amino-terminus (Asp1) and encompasses the amino acid sequence of an N-terminally elongated Aβ_−3–40_ peptide. Neuronal BE(2)-C cells and SH-SY5Y cells overexpressing APP were shown to be able to generate Aβ_−3–40_ in cell culture [23,25], and it appears that its cellular production occurs independently of BACE1 [25]. The self-assembly tendency of Aβ_−3–40_ was found to be lower than that of Aβ_1–42_ [23]. To the best of our knowledge, N-terminally elongated Aβ_−3–x_ peptides have not been identified in appreciable amounts in amyloid plaques, thus their potential significance in AD pathophysiology is currently unclear. The Aβ_−3–40_ (APP_669–711_) concentration in human blood plasma was reported to be essentially unchanged between subjects with or without amyloid-β positron-emission tomography (PET) imaging evidence of cerebral amyloid β accumulation. However, it was found to be a good reference to accentuate the selective pathological decrease in plasma Aβ_1–42_ in brain amyloid positive individuals. The plasma concentration ratio APP_669–711_/Aβ_1–42_ (Aβ_−3–40_/Aβ_1–42_) clearly outperformed plasma Aβ_1–42_ as a biomarker of brain amyloid deposition [22,23]. We report here on the characterization of a novel monoclonal antibody that was raised against the N-terminus of Aβ_−3–x_ and on the development and technical validation of a novel immunoassay for the measurement of Aβ_−3–40_ in biological samples. Finally, the relative levels of Aβ_−3–40_, Aβ_40_, and Aβ_42_ in human blood plasma in a small clinical sample were measured after pre-concentration by semi-automated Aβ magnetic bead immunoprecipitation. We observed statistically significant differences between patients with dementia of the Alzheimer’s type (AD-D) (*n* = 23) and dementia due to other reasons (OD) (*n* = 17) regarding Aβ_42_ and the concentration ratios Aβ_42_/Aβ_40_ and Aβ_42_/Aβ_−3–40_.

## 2. Results

### 2.1. Antibody Characterization 

**Figure 1:**
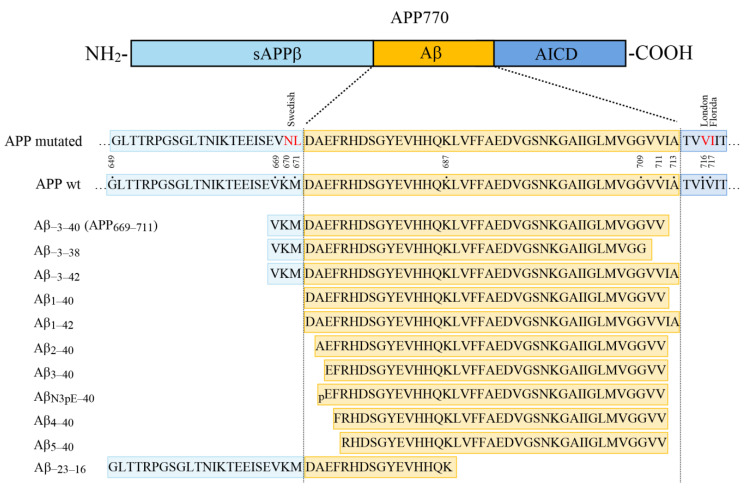
Amino acid sequences and numbering of the Aβ variants and amyloid precursor protein (APP)-related peptides that were studied. On top, a schematic diagram of the primary structure of the human APP770 isoform is shown. The Aβ peptide region, sAPPβ, and the APP intracellular domain (AICD) are indicated by different colours. Partial amino acid sequences of wild type APP770 and a mutant APP are shown in the middle panel. The positions of the Swedish (K670N/M671L) double mutation as well as the I716V (London) and V717I (Florida) mutations are highlighted in red colour. In the lower panel, the amino acid sequences and nomenclature of the 11 different synthetic peptides employed in this study are summmarized.

An overview of APP- and Aβ-related peptides employed in this study and the nomenclature used are shown in Figure 1.

**Figure 2:**
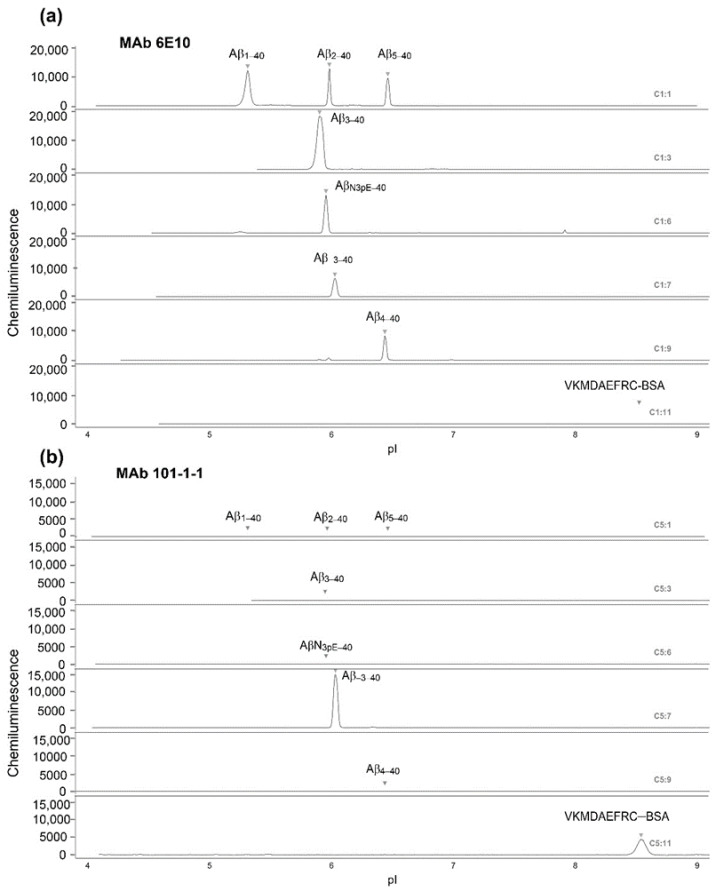
Monoclonal antibody 101-1-1 recognizes Aβ_−3–40_ with high selectivity on capillary isoelectric focusing immunoassay. A series of synthetic Aβ peptides with different N-termini was separated by isoelectric focusing in microcapillaries, immobilized by a photochemical reaction, and probed with (**a**) mAb 6E10 (2 µg/mL) or (**b**) mAb 101-1-1 (2.1 µg/mL). Chemiluminescent detection was achieved with biotinylated goat-anti mouse IgG antibody in combination with streptavidin-HRP (streptavidin horseradish peroxase conjugate) and chemiluminescent substrate. The specific Aβ variants loaded in the different capillaries as single peptides or in mixtures are indicated. Aβ_1–40_, Aβ_2–40_, Aβ_3–40_, Aβ_4–40_, Aβ_5–40_, and Aβ_−3–40_ were loaded at a concentration of 100 ng/mL. The tested concentration of Aβ_N3pE–40_ and VKMDAEFRC-bovine serum albumin (BSA) was 200 ng/mL.

The selectivity of the purified monoclonal antibody (mAb) 101-1-1 was assessed by capillary isoelectric focusing (CIEF) immunoassay, as described previously [8,26]. MAb 101-1-1 showed a strong signal with synthetic Aβ_−3–40_ (100 ng/mL), but not with Aβ_1–40_, Aβ_2–40_, Aβ_3–40_, Aβ_N3pE–40_, Aβ_4–40_, or Aβ_5–40_. MAb 101-1-1 also recognized the antigen VKMDAEFRC coupled to bovine serum albumin (BSA) (Figure 2).

**Figure 3:**
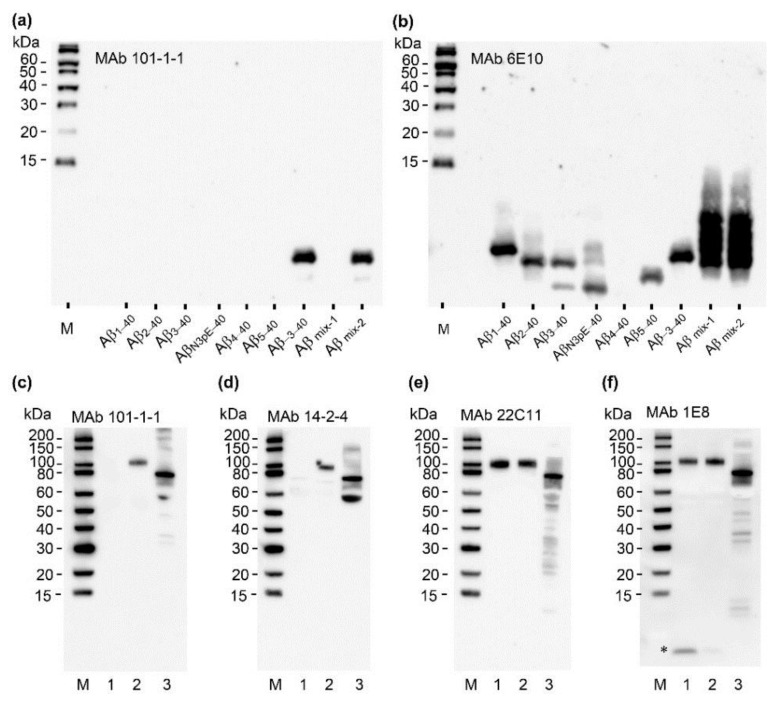
Western blot analysis of antibody selectivity. Upper panel: (**a**) The indicated synthetic Aβ peptides with different N-termini were separated on a 12%T/5% C urea-SDS-polycacrylamide gel, blotted onto PVDF, and probed with mAb 101-1-1 (1 µg/mL) in combination with goat anti-mouse IgG-peroxidase conjugate (1:30,000 dilution in PBS-T (phosphate buffered saline containing 0.075% Tween 20)). The peptide amounts loaded on the gel were 25 ng (Aβ_4–40_ and Aβ_5–40_) and 10 ng (remaining Aβ peptides), respectively. (M) Protein ladder; (Aβ mix-1) mixture containing 10 ng of each of Aβ_1–37_, Aβ_1–38_, Aβ_1–39_, Aβ_1–40_, and Aβ_1–42_; (Aβ mix-2) mixture containing 10 ng of each of Aβ_1–37_, Aβ_1–38_, Aβ_1–39_, Aβ_1–40_, Aβ_1–42_, and Aβ_−3–40_. The image was recorded after 5 min exposure. Image display: high: 65,535; low: 0; gamma: 0.6. (**b**) Reprobing of the same PVDF membrane without stripping with mAb 6E10 (1 µg/mL). Image recorded after 3 min exposure. Image display: high: 65,535; low: 0; gamma 0.61. Lower panel: Conditioned cell culture media of transfected H4 cells overexpressing Swedish mutant or wild type human APP751 and recombinant sAPPα (stock solution prepared in Diluent-35) were separated by SDS-polyacrylamide gel electrophoresis; blotted onto PVDF; and immuno-stained with (**c**) mAb 101-1-1, (**d**) mAb 14-2-4, (**e**) mAb 22C11, and (**f**) mAb 1E8. (M) Protein ladder; (1) 0.5 µL of cell culture supernatant H4APP751SWE; (2) 0.5 µL of cell culture supernatant H4APP751wt; and (3) 6.7 ng of recombinant sAPPα. Blot exposure times: mAb 101-1-1: 15 min, mAb 14-2-4: 6 min, mAb 22C11: 2 min, and mAb 1E8: 6 min. Image display (all four blots): max: 65,535; min: 0, gamma: 0.6). * Aβ (tentative assignment).

Essentially identical electropherograms indicating excellent selectivity for Aβ_−3–40_ were obtained with mAb 14-2-4 (anti Aβ_−3–X_, IBL International, Hamburg, Germany) (Figure S1). In the next step, the selectivity of mAb101-1-1 in Western blot detection of Aβ peptide variants separated under strongly denaturing conditions by urea-SDS-polyacrylamide gel electrophoresis (urea SDS-PAGE) was assessed. Again, mAb 101-1-1 showed excellent selectivity for synthetic Aβ_−3–40_. Under the tested conditions, no signals were observed with Aβ variants starting with Asp (1), Ala (2), Glu (3), pyroGlu (3), Phe (4), or Arg (5) (Figure 3a,b). Very similar results were obtained with mAb 14-2-4 (Figure S2). To evaluate whether the monoclonals 101-1-1 and 14-2-4 require the free N-terminal valine residue or also recognize APP or APP fragments starting N-terminal to the canonical Aβ sequence, we tested recombinant sAPPα and cell culture supernatants of H4 cells transfected with human wild type APP751 or Swedish mutant APP751SWE (K670N/M671L). Aliquots of cell culture supernatants and recombinant sAPPα were separated on a 4–12% Bis-Tris gradient gel, blotted onto PVDF (polyvinylidene difluoride membrane), and probed with mAb 101-1-1 or mAb 14-2-4 in combination with peroxidase labeled goat anti mouse IgG secondary antibody. For comparison, additional parallel blots were probed with mAb 22C11 (anti-APP) and mAb 1E8 (anti Aβ N-terminus). Both mAb 101-1-1 and 14-2-4 clearly detected recombinant sAPPα and secrected sAPP in cell culture supernatant of H4 cells overexpressing human wild type APP751, but not in conditioned medium of H4 cells overexpressing Swedish mutant APP (Figure 3c–f).

**Figure 4:**
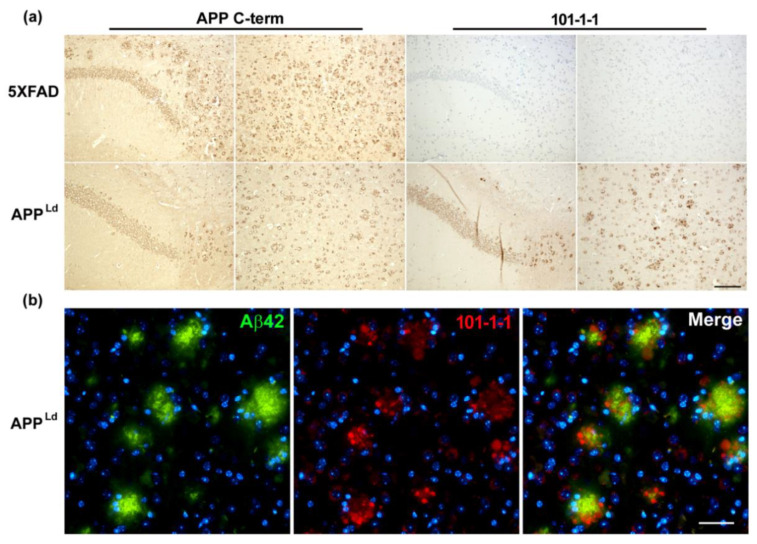
Immunohistochemical staining of Alzheimer’s disease (AD) transgenic mouse models. (**a**) Staining using an antibody directed against the carboxyterminus of APP (APP C-term) showed abundant cellular and neuritic immunoreactivity in 5XFAD and APP^Ld^ mice (left). In contrast, 101-1-1 showed an abundant staining only in APP^Ld^ mice harboring APP with an aminoterminal wildtype (K670–M671) sequence, but a complete lack of immunoreactivity in 5XFAD mice overexpressing APP with the Swedish mutation (N670–L671) (right). (**b**) Double-staining using an Aβ_42_-specific antibody (D3E10, green) and 101-1-1 (red) revealed abundant Aβ immunoreactivity in the central amyloid plaque cores, while the 101-1-1 signal was restricted to surrounding dystrophic neurites. Scale bar: 100 µm.

The recognition of wild type APP, but not APP carrying the Swedish double mutation KM/NL (670/671) by mAb 101-1-1 was further confirmed by immunohistochemistry (Figure 4). MAb 101-1-1 clearly stained cellular APP in brain slices from APP^Ld^ transgenic mice, but not in brain slices from 5XFAD transgenic mice. The latter express human APP and Presenilin 1 genes with a total of five mutations linked to AD, including the APP SWE double mutation. Double staining of brain slices of APP^Ld^ mice with anti Aβ_42_ (mAb D3E10) and mAb 101-1-1 indicated Aβ_42_ to be located in amyloid plaque cores, as expected. These were not recognized by mAb 101-1-1. Instead, mAb 101-1-1 signals were observed in close vicinity to the extracellular plaque cores, presumably representing APP accumulation within dystrophic neurites.

Next, the suitability of mAb 101-1-1 for magnetic bead immunoprecipitation (IP) after covalent coupling to Dynabeads M270 Epoxy was tested. Western blot analysis revealed successful IP of sAPP from conditioned media of H4APP751wt cells (Figure S3). After extended exposure, a faint peptide band co-migrating with synthetic Aβ peptides Aβ_−3–40_ and Aβ_1–40_ was also detected.

### 2.2. Immunoassay Development and Technical Validation

**Figure 5:**
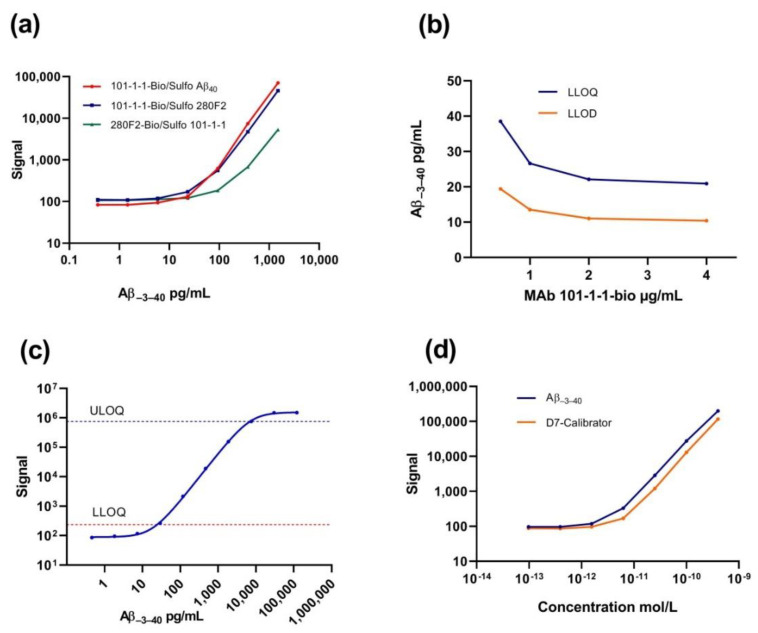
Assessment of different antibody combinations, capture antibody titration, quantitative assay range, and comparison of alternative calibrators. (**a**) Comparison of sandwich immunoassay variations employing mAb 101-1-1-biotin or 280F2-biotin for capture and SULFO-TAG Aβ_40_, SULFO-TAG 280F2, or SULFO-TAG 101-1-1 for detection. The tested antibody combinations are indicated. (**b**) lower limit of detection (LLOD) and lower limit of quantification (LLOQ) of the Aβ_−3–40_ immunoassay as functions of the 101-1-1-biotin capture antibody concentration. (**c**) Determination of the quantitative assay range. Upper limit of quantification (ULOQ) and LLOQ were calculated from the signals obtained with an extended fourfold calibrator dilution series plus blank (zero calibrator). (**d**) Comparison of dose–response curves obtained with synthetic Aβ_−3–40_ and D7-Calibrator.

A highly selective sandwich immunoassay for measuring Aβ_−3–40_ (APP_669–711_) was developed on the Mesoscale Discovery (MSD) technology platform employing 96-well small spot streptavidin pre-coated plates. Aliquots of mAb 101-1-1 and the anti Aβ_40_ C-terminal mAb 280F2 were conjugated (initially on a small scale) with biotin or SULFO-TAG to serve as capture or detection antibodies, respectively. After blocking unspecific binding sites, selected wells of a 96-well small spot streptavidin plate were coated with biotinylated mAb 101-1-1 capture antibody. A series of fourfold dilutions of synthetic Aβ_−3–40_ in Diluent-35 (1500 pg/mL, 375 pg/mL, 93.75 pg/mL, 23.44 pg/mL, 5.86 pg/mL, 1.46 pg/mL, 0.37 pg/mL, and 0 pg/mL) was assessed. With both SULFO-TAG Aβ_40_ detection antibody (MSD) and SULFO-TAG mAb 280F2, reasonable dose–response curves were obtained. The lower limits of detection (LLODs) were calculated by the MSD Discovery Workbench software as the smallest analyte concentration generating a signal three standard deviations (3 × SD) above the zero calibrator (blank) (minimum error estimates activated). The observed LLODs of 24.6 pg/mL (SULFO-TAG Aβ_40_) and 22.7 pg/mL (SULFO-TAG 280F2) were very similar. The flipped assay design with biotinylated mAb 280F2 serving for capture and SULFO-TAG 101-1-1 for detection produced substantially lower signals and a considerably higher calculated LLOD of 72.1 pg/mL (Figure 5a). Thus, we decided to proceed with assay development using biotinylated mAb 101-1-1 for capture and SULFO-TAG Aβ_40_ for detection. Next, a fresh lot of biotinylated mAb 101-1-1 was prepared on a larger scale, starting from 1 mg of purified mAb 101-1-1. The mole to mole ratio of incorporated biotin to protein was estimated by HABA (4-hydroxyazobenzene-2-carboxylic acid) assay for biotin quantitation and BCA (bicinchoninic acid) assay for protein quantitation. We calculated incorporation of approximately 2.9 moles of biotin per mole of protein and IgG recovery of approximately 90%. To optimize the detection of Aβ_−3–40_, different concentrations of biotinylated mAb 101-1-1 capture antibody during plate coating were tested (capture antibody titration). We observed an appreciable improvement in the detection sensitivity by increasing the concentration of the capture antibody from 0.5 µg/mL to 1 µg/mL and 2 µg/mL. Further increasing the capture antibody concentration to 4 µg/mL had a negligible additional effect (Figure 5b). At this stage and based on the results obtained so far, we drafted a first version of a standard operating procedure (SOP, see below, Materials and Methods section) to be evaluated in detail in a partial “fit for purpose” assay validation campaign. A cartoon displaying the assay principle is shown in Figure S4.

#### 2.2.1. Lower Limit of Detection, Lower Limit of Quantification, and Upper Limit of Quantification

The data analysis in each assay run included standard curve calculations with the MSD Discovery Workbench 4.0.12 software by four-parameter logistic curve fitting of raw signals obtained with seven or more fourfold serial dilutions of the synthetic Aβ_−3–40_ calibrator peptide plus a zero calibrator (blank). The lower limit of detection (LLOD) was calculated for each single assay plate as the lowest analyte concentration generating a signal three SDs above the lowest standard/blank with the option “use minimum error estimates” activated. The lower limit of quantification (LLOQ) was defined as the lowest analyte concentration producing a signal 10 SDs above the lowest standard/blank. Mean LLOD and LLOQ calculated from *n* = 5 assay runs within the validation study were 11.04 pg/mL ± 0.44 and 22.24 pg/mL ± 1.36 (mean ± SD), respectively. To assess the upper limit of quantification (ULOQ), on a single assay plate, an extended fourfold calibrator dilution series including ten fourfold serial dilutions starting at 120 ng/mL plus a zero calibrator/blank was analyzed. The ULOQ was defined as the highest calibrator concentration that showed < 20% coefficient of variation (CV) between two technical replicates (regarding both signal and calculated concentration) and 85–115% recovery (i.e., the calculated concentration had to be in the range of 85–115% of the theoretical calibrator concentration). The observed ULOQ in this experiment was 7.5 ng/mL (Figure 5c).

#### 2.2.2. Alternative Calibrator

Aβ peptides are notoriously prone to aggregation. We thus evaluated a synthetic molecule (D7-Calibrator) comprising the Aβ_−3–X_ N-terminal amino acid sequence VKMDAEFRH followed by 5x PEG2 (8-Amino-3,6-dioxaoctanoic acid) and the Aβ_40_ C-terminus GLMVGGVV (Mr: 2571) (Biosyntan, Berlin, Germany) as a potential alternative calibrator. The D7-Calibrator was designed to provide improved water-solubility and to be less prone to aggregation or adsorption. The physicochemical properties have not been studied in detail. A stock solution (3.05 mg/mL) was prepared in H_2_O, and further dilutions were done in Diluent-35. Fourfold serial dilutions of the D7-Calibrator starting with 1029 pg/mL produced a reasonable standard curve with an LLOD of 11.2 pg/mL (Figure 5d). To compare dose–response curves, taking into account the difference in the molecular masses of Aβ_−3–40_ (Mr = 4685.4) and the D7-Calibrator (Mr = 2571), the x-axis of the plot shown in Figure 5d was scaled in moles/L. At equal molar concentrations, Aβ_−3–40_ produced slightly higher signals than the D7-Calibrator. Nevertheless, the findings suggest that the D7-Calibrator provides an optional alternative standard/calibrator to the full length Aβ_−3–40_ peptide.

#### 2.2.3. Technical Intra-Assay Repeatability and Spatial Bias

To check for potential positional effects (spatial bias), synthetic Aβ_−3–40_ was diluted in Diluent-35 to a final concentration of 469 pg/mL and measured in 24 technical replicates distributed across one 96-well assay plate. The coefficient of variation in terms of the measured signals was 7.6%. The mean calculated concentration was 485.8 pg/L and the coefficient of variation (%CV) between calculated Aβ_−3–40_ concentrations in the 24 replicates was 4.7%. Thus, in general, the technical repeatability of the assay was good. However, in position H12 (lower right corner of the assay plate), the measured signal was 23% lower than the mean signal. To exclude a systematic positional effect (spatial bias) of our system, on a second assay plate, we tested 11 technical replicates of a corresponding Aβ_−3–40_ dilution. The observed %CVs were 11.1% (measured signal) and 7.1% (calculated concentrations). Importantly, on this assay plate, the measured signal in position H12 was 10.3% above the mean signal, suggesting random error and arguing against systematic spatial bias. Heat maps indicating the distribution of the measured signals are shown in Figure S5.

#### 2.2.4. Cross Reactivity/Assay Specificity

Next, we assessed the cross reactivity of the sandwich immune assay with related Aβ peptide variants and sAPPα. To that end, Aβ_1–40_, Aβ_1–42_, Aβ_−3–38_, and Aβ_−3–42_ were tested at concentrations of 4 ng/mL and 20 ng/mL and recombinant sAPPα at a concentration of 320 ng/mL. Aβ_1–40_, Aβ_1–42_, and Aβ_−3–38_ measurements at both tested concentrations were below the detection range of the assay plate. Aβ_−3–42_ produced signals within the detection range. The calculated concentrations were 12.5 pg/mL when 4 ng/mL of Aβ_−3–42_ was applied and 51.6 pg/mL when 20 ng/mL of Aβ_−3–42_ was analyzed. Thus, approximately 0.3% of the Aβ_−3–42_ peptide was recognized in the Aβ_−3–40_ assay, indicating a cross reactivity <0.5%. The signal observed with 320 ng/mL of recombinant sAPPα was below the detection range of the assay (Table 1).

#### 2.2.5. Between-Run Variability/Inter-Assay Variance of the Aβ_−3–40_ Immunoassay

The initial core technical assay validation campaign included five independent runs of the Aβ_−3–40_ chemiluminescence immunoassay that were executed within eight weeks. On each of the five assay plates, an aliquot of a quality control (QC) sample was measured in four technical replicates. The primary intended application of the novel assay was to measure Aβ_−3–40_ after pre-analytical enrichment of Aβ peptides from EDTA-blood plasma by magnetic bead immunoprecipitation (IP) [21]. A QC-IP-eluate was prepared by running several IPs in parallel from pooled human blood plasma and combining the eluates after 4.8-fold dilution with Diluent-35 to a single QC-IP-eluate. Aliquots for single use were stored at −80 °C until analysis. The results obtained with the QC-IP-eluate in five independent assay runs are summarized in Table 2. The electro-chemiluminescence signals observed in the five different experiments (means of four technical replicates on each assay plate) ranged from 6755 to 8216, with a coefficient of variation of 8.2%. The mean calculated concentration of Aβ_−3–40_ in the QC-IP-eluate was 217 pg/mL, with a coefficient of variation of 16.3%, which was outside of the predefined acceptance range of 15% CV (Table 2). The relatively large scatter in terms of the calculated concentration of Aβ_−3–40_ was apparently caused by assay run #4, which seemed to differ substantially from the remaining four experiments and was identified as an outlier by Grubbs test (α = 0.05). Notably, the serial calibrator dilutions for assay run #4 had been prepared from a higher starting concentration, and thus following a slightly different experimental protocol than for the remaining experiments. This was done in order to allow for determination of the ULOQ on assay plate #4 (see above). Excluding plate #4 from the analysis reduced the CV of the measured concentrations to 4.7%. Taken together, the findings suggest that a strict standard operation procedure (SOP) covering all experimental steps including the preparation of calibrator dilutions needs to be implemented. Furthermore, a suitable QC sample should be included to allow for normalization between assay plates (see below).

#### 2.2.6. Impact of Pre-Analytical Sample Handling Procedures

**Figure 6:**
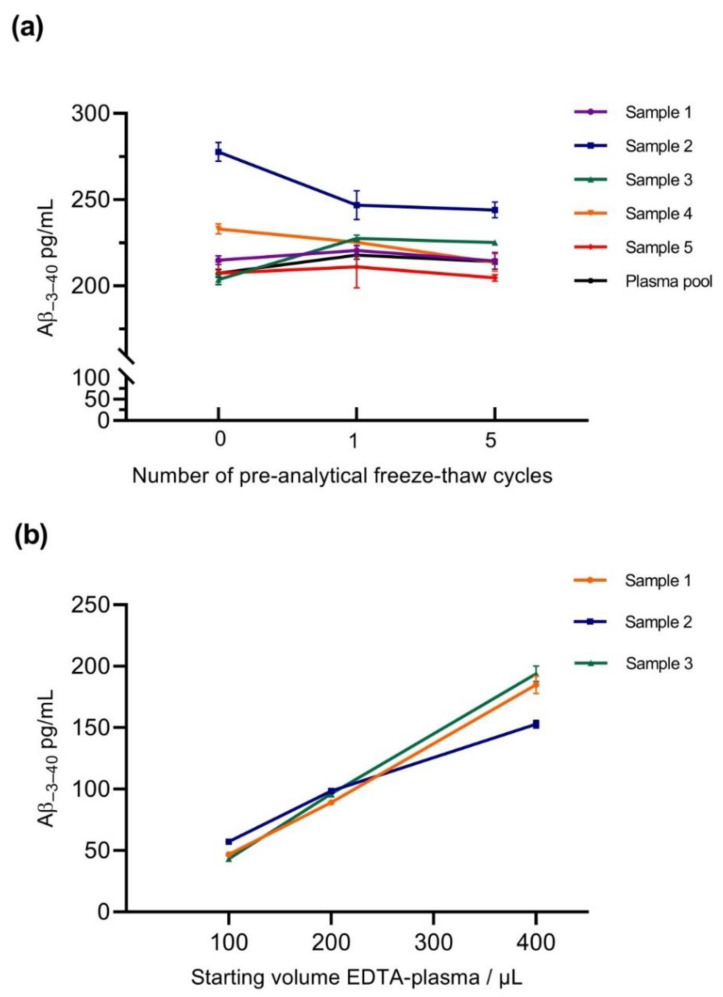
Impact of pre-analytical sample handling procedures on the measurement of Aβ_−3–40_. (**a**) Impact of pre-analytical freezing and thawing of diluted IP eluates. IP eluates obtained by 1E8 magnetic bead IP from aliquots of five individual and one pooled plasma sample were diluted with Diluent-35 and measured in the Aβ_−3–40_ assay freshly or after one or five freeze–thaw cycles. Mean calculated concentrations from two technical replicates on the assay plate and SDs are shown. (**b**) Impact of changing the starting volume and diluting the blood plasma used for IP. IPs from three different original blood plasma samples were done starting from 400 µL of EDTA plasma, from 200 µL of EDTA plasma mixed with 200 µL of Diluent-35, and from 100 µL of EDTA-Plasma plus 300 µL of Diluent-35. The resulting IP eluates were diluted 4.8-fold with Diluent-35 and stored at −80 °C until the analysis. Means ± SDs from triplicate reads are shown.

Aliquots of individual EDTA-blood plasma samples from five different donors and a pooled plasma sample were subjected to magnetic bead immunoprecipitation with mAb 1E8. Aβ-peptides were eluted by heating in 20mM bicine, 0.6% 3-((3-cholamidopropyl) dimethylammonio)-1-propanesulfonate, pH 7.6 (bicine-CHAPS), as described previously [26], and diluted 4.8-fold with Diluent-35 [21]. The diluted IP eluates were measured with the Aβ_−3–40_ assay freshly or after single or multiple freezing and re-thawing. We did not observe a substantial systematic impact of single or multiple freezing and thawing of the diluted IP eluates on the measured concentrations of Aβ_−-3–40_ (Figure 6a). A separate and independent control experiment did not suggest a general and systematic impact of a single or five repeated freeze–thaw cycles on the measurement of Aβ_38_, Aβ_40_, and Aβ_42_ by the V-Plex Aβ panel 1 (6E10) multiplex assay kit (Mesoscale Discovery, MSD) either (Figure S6). Consequently, pre-analytical freezing of IP eluates after dilution in Diluent-35 was implemented in the SOP. To assess the impact of reducing the blood plasma volume and dilution with Diluent-35 prior to IP, three different EDTA-plasma samples were studied. IPs were executed starting from 400 µL of EDTA plasma, from 200 µL of EDTA plasma mixed with 200 µL of Diluent-35, and from 100 µL of EDTA-Plasma mixed with 300 µL of Diluent-35. In each case, 100 µL of 5× triple detergent IP-buffer concentrate and 25 µL of 1E8 magnetic beads were added for overnight incubation. IP eluates were diluted 4.8-fold with Diluent-35 and stored at −80 °C until the analysis. As expected, after reducing the starting volume of EDTA-plasma and diluting the samples with Diluent-35, lower concentrations of Aβ_−3–40_ were measured in the IP eluates. In two out of the three tested blood plasma samples, the measured Aβ_−3–40_ concentrations decreased in a linear fashion with sample dilution (Figure 6b). In the remaining sample, we observed a slight relative increase in Aβ_−3–40_ recovery after sample dilution, suggesting the presence of interfering compounds (matrix effects) in undiluted EDTA-plasma.

#### 2.2.7. Specificity of the Two-Step Immunoassay

To further evaluate the specificity of the two-step immunoassay for measuring Aβ_−3–40_ in human blood plasma, a number of control IPs were done and analyzed by the Aβ_−3–40_ immunoassay. The signals we obtained after magnetic bead immunoprecipitation from pooled EDTA-plasma using the anti-human Tau monoclonal HT7 and after 1E8-IP from 1% BSA in phosphate buffered saline (PBS) (“mock IP”) were below the detection range. The observations indicate absence of appreciable background signal owing to unspecific binding of plasma components to magnetic beads functionalized with an unrelated monoclonal antibody or from the 1E8-magnetic beads per se. Next, an Aβ-depleted plasma sample was prepared by two consecutive rounds of Aβ-IP from pooled plasma with a mixture of magnetic beads coupled with anti Aβ monoclonals 6E10 or 4G8. The measured concentrations of Aβ_−3–40_ were 12.3 pg/mL in the Aβ-depleted sample and 239.3 pg/mL in the corresponding control sample, indicating 95% reduction in Aβ_−3–40_ after Aβ depletion (Table 3).

#### 2.2.8. Assay Interference

In an additional and separate control experiment, we assessed to what extent sAPPα, a related synthetic model peptide (Aβ_−23–16_), and Aβ_1–40_ interfered with the measurement of Aβ_−3–40_ in a diluted IP eluate from pooled human plasma. The model peptide Aβ_−23–16_ (APP_649–687_) (APP_770_ numbering) comprised 23 amino acids of the APP sequence N-terminal to the Aβ sequence followed by Aβ_1–16_. Both sAPPα and Aβ_−23–16_ are recognized by mAb101-1-1. The addition of sAPPα or Aβ_−23–16_ reduced the measured Aβ_−3–40_ concentration in comparison with the control in a dose-dependent fashion (Table 4). In contrast, the addition of Aβ_1–40_ affected the measurement of Aβ_−3–40_ only marginally, even at the highest tested concentration of 45 ng/mL. The experiment was repeated once with a comparable outcome (data not shown). Notably, assay interference by sAPPα or related APP fragments is not expected to represent a serious issue in the Aβ_−3–40_ two-step immunoassay, because the described magnetic bead IP with a covalently immobilized antibody against the Aβ N-terminus followed by heat elution in bicine-CHAPS pre-concentrates Aβ peptides very efficiently [21,26]. However, high concentrations of sAPP or related APP derivatives might interfere with the correct direct measurement of absolute Aβ_−3–40_ concentrations in biological samples, dependent on the type of sample that is analyzed.

#### 2.2.9. Efficiency of the Aβ_−3-–40_ Immunoprecipitation by 1E8-Magnetic Beads

To estimate how efficiently Aβ_−3–40_ is immunoprecipitated under the tested experimental conditions, we performed two consecutive rounds of 1E8 IP from a pooled plasma sample. Comparison of the measured Aβ_−3–40_ concentrations in the IP eluates of the first and second IP round indicated high recovery after a single round of IP (Table 5). The measured Aβ_−3–40_ concentration after the second IP was reduced to approximately 7.5% of that observed in the eluate obtained after the first IP.

#### 2.2.10. Analytical Spike Recoveries

Two different individual EDTA plasma samples and a pooled plasma sample were spiked with synthetic Aβ_−3–40_ at three different spike levels (10 pg, 20 pg, or 40 pg spiked into 400 µL of plasma) prior to immunoprecipitation with 1E8-magnetic beads. The diluted IP eluates obtained from neat and spiked samples were measured with the Aβ_−3–40_ assay. Spike recoveries were calculated by comparing the amount of the spike added (in pg) with the amount detected in the spiked samples after subtraction of the endogenous Aβ_−3–40_ peptide found in the neat sample. The observed spike recoveries are summarized in Table 6. Spike recoveries ranged from 32.5% to 99.6%, with mean values of 76% at the low spike level (10 pg of Aβ_−3–40_ added to 400 µL of EDTA plasma), 94% at the intermediate spike level (20 pg of Aβ_−3–40_ added to 400 µL of EDTA plasma), and 67% at the high spike level (40 pg of Aβ_−3–40_ added to 400 µL of EDTA plasma). The findings suggest a certain degree of matrix interference, which appears to vary between biological samples. In a control IP from 400 µL Diluent-35 spiked with 40 pg of Aβ_−3–40_, we observed 91.7% spike recovery.

#### 2.2.11. Repeatability of Manual IPs from the Same Sample Performed in Parallel

Three different original EDTA plasma samples were studied. From each, three immunoprecipitations were executed in parallel, resulting in nine IP eluates in total. The observed coefficient of variation between the parallel IP reactions in terms of measured Aβ_−3-–40_ concentrations was <10% (Table 7).

#### 2.2.12. Day-to-Day Variance of the Manual IP Procedure

On four different days within 2 weeks, 1E8-magnetic bead IPs were executed from aliquots of five different human EDTA-plasma samples (termed A, B, C, D, E). The IP eluates obtained on each of the four days were diluted with Diluent-35 and stored frozen at −80 °C. Finally, all 20 samples were thawed and measured on a single assay plate. The coefficients of variation regarding the measured Aβ_−3–40_ concentrations in IP eluates prepared on four different days ranged from 3.6 to 20.1%, with a mean value of 9.1% (Table S2).

#### 2.2.13. Intra-Assay Variance/Repeatability of Aβ_−3–40_ Measurements in IP-Eluates

Diluted IP eluates obtained from three different original EDTA plasma samples were measured on the same assay plate in eight technical replicates each. The observed coefficients of variation between the replicate measurements were 2.2%, 2.7%, and 2%, respectively (Table S1).

#### 2.2.14. Day-to-Day Variance of the Complete Two-Step Immunoassay and Normalization

To evaluate the intermediate imprecision (day-to-day variance) of the complete two-step immunoassay procedure (manual IP followed by immunoassay), we tested a set of three individual control plasma samples and one control plasma pool in four different experiments. In each of these four assay runs, separate immunoprecipitations were performed from aliquots of the control samples. The measured Aβ_−3–40_ concentrations varied substantially between the four experiments, with a mean coefficient of variation of 29.6% (Table 8). Both the IP and the subsequent Aβ_−3–40_ immunoassay are expected to contribute to the overall intermediate imprecision (see above). In the next step, we normalized the measured Aβ_−3–40_ concentrations by a QC-IP eluate included on each assay plate (see above). It served to calculate assay-plate correction factors for normalization of the error contributed by the four independent runs of the Aβ_−3–40_ immunoassay only. The overall mean coefficient of variation of the complete two-step immunoassay after normalization was 16.2%. This was within the predefined acceptance range (<20% CV), but still larger than the desired variance of <15% CV.

### 2.3. Measurement of Aβ_−3–40_ in Patient Samples and Determination of Aβ Ratios as AD Biomarker Candidates

The relative levels of Aβ_−3–40_, Aβ_40_, and Aβ_42_ were measured in 40 human blood plasma samples from patients with dementia of the Alzheimer’s type (AD-D, *n* = 23) and other dementias (OD, *n* = 17) by a semi-automated version of our two-step immunoassay. Here, the 1E8-magnetic bead IPs were executed on a CyBio FeliX liquid handling instrument (Analytik Jena GmbH, Jena, Germany) starting from 200 µL of EDTA-blood plasma mixed with 200 µL of H_2_O. The resulting IP eluates were diluted 4.8-fold with Diluent-35 and stored in aliquots at −80 °C until they were measured with the novel Aβ_−3–40_ assay and the V-Plex Aβ panel 1 (6E10) multiplex assay kit. Importantly, the clinical cohort (*n* = 40 subjects) studied here is the same we investigated previously using our original two-step immunoassay procedure including Aβ pre-concentration by manual IP starting from 400 µL of EDTA-plasma [21]. In all 40 samples, Aβ_−3–40_, Aβ_40_, and Aβ_42_ measurements were in the detection range of the assay. Baseline statistics are summarized in Table S3. The measured concentrations of Aβ_−3–40_, Aβ_40_, and Aβ_42_, as well as the concentration ratios Aβ_42_/Aβ_−3–40_ and Aβ_42_/Aβ_40_, showed lognormal distributions (Table S4). Thus, we performed pairwise comparisons of means between the diagnostic groups OD (*n* = 17) and AD-D (*n* = 23) by multiple t-tests on log2-transformed data. The *p*-values were corrected for multiple comparisons using the Holm–Sidak method with α = 0.05 and without assuming a consistent SD. There were no statistically significant differences between the diagnostic groups OD and AD-D regarding the mean concentrations of Aβ_−3–40_ and Aβ_40_ (log2-transformed data). Aβ_42_, the Aβ_42_/Aβ_40_ ratio, and the Aβ_42_/Aβ_−3–40_ ratio (log2 transformed data) were statistically significantly lower in the AD-D patients (Table 9).

**Figure 7:**
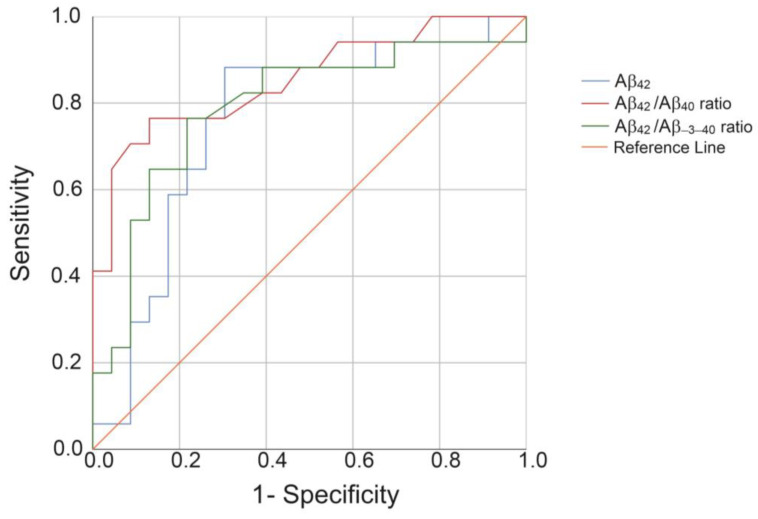
Receiver operating characteristic (ROC) analysis of Aβ_42_, the Aβ_42_/Aβ_40_ ratio, and the Aβ_42_/Aβ_−3–40_ ratio as discriminators of patients with other dementias (OD) and Alzheimer’s type dementia (AD-D). The ROC curves of the indicated biomarker candidates in blood plasma are shown in discriminating the diagnostic groups OD and AD-D. The areas under the curves (AUCs) were 0.760 (Aβ_42_), 0.790 (Aβ_42_/Aβ_−3–40_ ratio), and 0.854 (Aβ_42_/Aβ_40_ ratio), respectively.

The diagnostic potential of plasma Aβ_42_ and the Aβ_42_/Aβ_40_ and Aβ_42_/Aβ_−3–40_ ratios for the discrimination between OD versus AD-D in this clinical sample was compared by receiver operating characteristic (ROC) analyses. The areas under the ROC curves (AUCs) were 0.760 for Aβ_42_, 0.790 for the Aβ_42_/Aβ_−3–40_ ratio, and 0.854 for the Aβ_42_/Aβ_40_ ratio (Figure 7).

## 3. Discussion

Affordable blood-based surrogate biomarker assays that can reliably detect AD associated pathological changes at an early disease stage have enormous potential to support early and preclinical diagnosis and the development of novel, disease modifying treatments. Recent studies have shown that specific forms of the Aβ peptide can be detected in human blood plasma and represent highly promising AD biomarker candidates. For example, a multimer detection system employing two antibodies with overlapping N-terminal Aβ-epitopes for measuring Aβ oligomerization after spiking synthetic Aβ into blood plasma samples has been developed [27]. Correlations between the plasma oligomerized Aβ and CSF Aβ_42_, amyloid-positron-emission tomography (PET) results [28], and measures of cognitive functions [29,30] were reported. Other research groups discovered that the plasma ratios Aβ_42_/Aβ_40_, Aβ_1–40_/Aβ_1–42_, and APP_669–711_/Aβ_1-42_ (i.e., Aβ_−3–40_/Aβ_1–42_), as measured by immunoprecipitation followed by mass spectrometry, predicted brain amyloid pathology with high accuracy [19,20,23]. To the best of our knowledge, so far, commercial antibodies against N-terminally elongated Aβ_−3–X_ or immunoassays for the specific measurement of Aβ_−3–40_ have not been available.

To set the groundwork for studying the biology of Aβ_−3–40_ in more detail in future studies and for assessing Aβ_−3–40_ as a potential reference for accentuating the AD-associated selective decrease in Aβ_42_ in blood plasma and possibly other biological fluids, we have characterized two novel antibodies raised against the Aβ_−3–X_ N-terminus and developed a highly selective sandwich immunoassay for measuring Aβ_−3–40_. Our observations show that the monoclonal antibodies 101-1-1 and 14-2-4 can detect Aβ_−3–40_ without appreciable cross-reactivity with the canonical Aβ_1–40_ or N-terminally truncated Aβ-variants in CIEF immunoassay and urea-SDS-PAGE/Western blot analysis. As it turned out, neither of the two antibodies are specific for the free N-terminal valine. Both also recognize amyloid precursor protein (APP), provided it does not carry the Swedish (K670N/M671L) double mutation located directly N-terminal to the β-secretase cleavage site. Whether or not the mAbs 101-1-1 and 14-2-4 display preferences for monomeric, oligomeric, or aggregated forms of the N-terminally elongated Aβ_−3–40_ has not been assessed explicitly here. Published in vitro experiments including Thioflavin-T aggregation assay, size exclusion chromatography, and circular dichroism suggested a substantially lower self-aggregation tendency of Aβ_−3–40_ than Aβ_1–42_ [23]. To the best of our knowledge, Aβ_−3–40_ has not been reported in amyloid plaques so far, and essentially no information regarding its potential role in AD pathogenesis is available. In brain slices from APP^Ld^ transgenic mice analyzed in our present study, mAb 101-1-1 appeared to bind neuronal cell bodies and to dystrophic neurites resembling abnormal neuronal processes, but not to plaque cores. Presumably, the observed signals represent cellular APP.

MAb 101-1-1 was selected to serve as the capture antibody in the finalized version of our novel Aβ_−3–40_ immunoassay. The LLOD of the assay for measuring Aβ_−3–40_ was approximately 11 pg/mL, and the quantitative assay range was approximately 22 pg/mL–7.5 ng/mL. We did not observe appreciable cross reactivity with Aβ_1–40_, Aβ_1–42_, Aβ_−3–38_, and sAPPα. In contrast, at high concentrations, synthetic Aβ_−3–42_ produced measurable signals, indicating low cross reactivity at a level of <0.5%. The technical intra-assay repeatability passed the predefined acceptance limit of <15% CV, and we did not find evidence for systematic spatial bias. The inter-assay CV of the calculated Aβ_−3–40_ concentration in a QC IP eluate that was measured in five independent experiments within the assay validation campaign was 16.3%, and thus outside of the predefined 15% CV acceptance range. It appeared that the large scatter was owing to a single experiment in which the calibrator dilution series had been prepared in a slightly deviant way (to allow for determination of the ULOQ in this particular assay run). Accordingly, we conclude that a strict SOP covering all pre-analytical and analytical steps in detail has to be implemented to minimize inter-assay variability.

The primary intended purpose of the novel assay is the assessment of Aβ_−3–40_ and the Aβ_42_/Aβ_−3–40_ ratio (or reverse) in human blood plasma as a candidate biomarker of AD. In view of published observations from IP mass spectrometry studies [22,23,24], the blood concentration of Aβ_−3–40_ was expected to be small. Thus, we decided to pre-concentrate Aβs from plasma by magnetic bead immunoprecipitation and to analyze the Aβ_−3–40_ plasma level by a two-step immunoassay procedure, essentially as described previously for the measurement of Aβ_38_, Aβ_40_, and Aβ_42_ [21]. A control experiment with a pooled blood plasma sample confirmed high yield of Aβ_−3–40_ after a single round of 1E8 magnetic bead IP. Pre-analytical freezing and thawing of diluted IP eluates did not seem to substantially and systematically affect the measurement of Aβ_−3–40_. Consequently, freezing of the diluted IP eluates at −80 °C was implemented in our two-step immunoassay protocol to completely separate pre-analytical Aβ-enrichment from the actual measurement, and thus facilitate the execution of the two-step immunoassay under routine laboratory conditions. Furthermore, this allowed for testing a semi-automated prototypic IP procedure executed at a different site (see below). We have shown that the two-step immunoassay for Aβ_−3–40_ is highly selective. We did not observe appreciable signals in plasma IP eluates from magnetic bead IP with an unrelated antibody or from a simulated IP with 1E8 magnetic beads. Thus, unspecific binding of other plasma components to functionalized anti mouse IgG magnetic beads or background signal directly stemming from the 1E8 coupled beads could be excluded. Depleting Aβ by two rounds of IP with anti Aβ mAbs 6E10 plus 4G8 prior to the 1E8 IP reduced the measured Aβ_−3–40_ concentration by approximately 95%.

Analytical spike recoveries in the two-step immunoassay varied substantially between the tested plasma samples and different spike levels indicating matrix interferences. Thus, the measured Aβ_−3–40_ levels in the diluted IP eluates do not allow for calculating the true, absolute concentrations of this Aβ peptide in plasma samples, but have to be considered relative.

The intermediate imprecision (between-run variance) of the complete manual two-step immunoassay reached an acceptable CV of 16.2% only after normalization. Notably, in this case, the normalization addressed the technical error contributed by the Aβ_−3–40_ assay only, not the scatter of the manual IP procedure. Without normalization, the mean between-run CV of the measured Aβ_−3–40_ concentrations in four QC-samples was 29.6%, and thus clearly above the predefined acceptance criterion of <20% CV. In conclusion, appropriate QC samples allowing for normalization should be included in future experiments, whenever possible, and further technical improvement is desirable. 

To finally test the novel Aβ_−3–40_ immunoassay on biological samples in a pilot study and to demonstrate the possibility of automation of the magnetic bead IP, we studied the same small clinical sample we have previously described in detail [21]. In our current study, Aβ was pre-concentrated from 1:2 dilutions of blood plasma with water by a prototypic semi-automated 1E8 magnetic bead IP protocol. The IP eluates were diluted 4.8-fold with Diluent-35 and frozen in aliquots for single use at −80 °C. For measuring Aβ_−3–40_, Aβ_40_, and Aβ_42_ at a different site, the samples were transported on dry ice. We did not observe a statistically significant difference in the mean (log2 transformed) Aβ_−3–40_ levels between patients with AD-D (*n* = 23) and OD (*n* = 17). In contrast, Aβ_42_ and the Aβ ratios Aβ_42_/Aβ_−3–40_ and Aβ_42_/Aβ_40_ were statistically significantly lower in patients with AD-D compared with those in the OD group. These findings are in agreement with the IP mass spectrometry data collected in two independent cohorts by Nakamura et al. [23]. The areas under the ROC curves in our small data set were 0.76 (Aβ_42_), 0.79 (Aβ_42_/Aβ_−3–40_ ratio), and 0.85 (Aβ_42_/Aβ_40_ ratio). These observations support the idea that a suitable reference may serve to accentuate the pathological AD-associated decrease in soluble Aβ_1–42_ [22] (or Aβ_42_, respectively). Furthermore, our findings show that a prototypic semi-automated pre-analytical IP protocol works and that the adaptions and modifications to the original manual two-step immunoassay that were implemented did not lead to masking or loss of the reduced Aβ_42_/Aβ_40_ plasma ratio as a peripheral diagnostic biomarker of AD. Further studies employing independent and larger clinical cohorts will be required to assess the diagnostic potential of the two-step immunoassay for the Aβ_42_/Aβ_−3–40_ ratio in detail. Preferably, healthy controls and patients in different stages of AD should be included

In summary, we have developed a novel, highly selective sandwich immunoassay for measuring Aβ_−3–40_ in biological samples. In combination with pre-analytical Aβ enrichment by magnetic bead IP, the assay can serve to measure the relative levels of Aβ_−3–40_ in human blood plasma. Thus, the methodological groundwork has been set for future studies addressing the diagnostic potential of the Aβ_42_/Aβ_−3–40_ ratio (or reverse) as a novel surrogate biomarker candidate of cerebral amyloid deposition.

## 4. Materials and Methods

Antibodies: The mouse monoclonal antibody (mAb) 101-1-1 was developed, produced, and purified by Biogenes GmbH (Berlin, Germany) on a fee for service basis. The synthetic peptide VKMDAEFRC-amide (Biosyntan, Berlin, Germany) was conjugated to bovine thyroglobulin as carrier protein and served for immunization of mice. Hybridoma cells were screened by ELISA using Aβ_1–40_ and Aβ_2–40_ for negative screening and VKMDAEFRC-BSA-conjugate for positive screening. Clone 101-1-1 was obtained after two rounds of cloning. We received 88 mg of purified monoclonal antibody mAb 101-1-1 for antibody characterization, assay development, assay validation, and further use. Monoclonal anti Aβ_−3–x_, clone 14-2-4, was obtained from IBL International/Tecan, (Hamburg, Germany). MAb 1E8 (anti Aβ N-terminus) and mAb 5C3 (anti Aβ_40_) were purchased from nanoTools GmbH (Teningen, Germany). MAb 280F2 (anti Aβ_40_) was obtained from Synaptic Systems (Goettingen, Germany), mAb 6E10 from Biolegend (San Diego, CA, USA), anti APP mAb 22C11 from Merck-Millipore (Darmstadt, Germany), and anti-Human Tau mAb HT7 from Thermo Scientific (Waltham, MA USA). The rabbit monoclonal anti Aβ_42_ antibody mAb D3E10 was purchased from Cell Signaling Technology (Danvers, MA, USA), while the rabbit polyclonal anti C-terminus APP A8717 was purchased from Sigma-Aldrich (Munich, Germany). SULFO-TAG Aβ_40_ detection antibody was obtained from Mesoscale Discovery, Rockville, MD, USA. Biotinylated horse anti-mouse IgG was purchased from Vector Laboratories (Burlingame, CA, USA), streptavidin-biotinylated peroxidase complex from GE Healthcare, Chicago, IL, USA), and goat anti mouse IgG peroxidase conjugate from Calbiochem/Millipore/Merck (Darmstadt, Germany). Biotinylated secondary anti-mouse and anti-rabbit antibodies, used in DAB-immunohistochemical staining, were obtained from DAKO/Agilent (Waldbronn, Germany). Fluorescent goat anti-Mouse IgG (DyLight 594) donkey anti-Rabbit IgG (DyLight 488) secondary antibodies were purchased from Thermo Scientific (Waltham, MA, USA).

Recombinant sAPPα was obtained from Sigma-Aldrich (Munich, Germany). Aβ_−3–40_, Aβ_−3–38_, and Aβ_−3–42_ were synthesized as described in detail previously [25]. The synthetic peptides Aβ_1–40_, Aβ_2–40_, Aβ_3–40_, Aβ_N3pE–40_, Aβ_4–40_, and Aβ_5–40_ were obtained from AnaSpec (Fremont, CA, USA). The synthetic model peptide Aβ_−23–16_ (APP_649–687_) (H_2_N-GLTTRPGSGLTNIKTEEISEVKMDAEFRHDSGYEVHHQK-CONH_2_) was synthesized using standard solid-phase fluorenylmethoxycarbonyl (Fmoc) chemistry. Quality control of the purified product was performed by reversed-phase high-performance liquid chromatography coupled to a single-quadrupole mass spectrometer (Alliance/QDa, Waters Corporation, Milford, MA, USA) and by high-resolution mass spectrometry using a MALDI-TOF/TOF mass spectrometer (UltrafleXtreme, Bruker, Billerica, MA, USA).

The CIEF immunoassay was performed as described before [8,26]. For chemiluminescence detection, goat anti-mouse IgG, streptavidin-peroxidase conjugate, and Luminol/peroxide (all 3 reagents obtained from ProteinSimple, San Jose, CA, USA) were employed.

Preparation of functionalized anti-Aβ magnetic beads: Dynabeads M280 sheep anti mouse IgG (ThermoFisher Scientific, Waltham, MA, USA) were functionalized by covalent coupling to mAb 1E8 according to the manufacturer’s instructions and as described before [21]. Dynabeads M-270 Epoxy (Invitrogen/ThermoFisher Scientific, Waltham, MA, USA) were covalently coupled to mAb 101-1-1 following the manufacturer’s instructions.

Antibody labeling: For small-scale biotinylation and SULFO-Tag labeling, 80 µL of a 1 mg/mL stock solution of mAb 101-1-1 or 280F2 was buffer-exchanged into MSD conjugation buffer (PBS, pH 7.9, preservative-free) on a Zeba Spin Desalting column 40 K MWCO, 0.5 mL (Thermo Scientific, Waltham, MA, USA). The buffer-exchanged antibody solution was divided into two halves, each containing approximately 40 µg of IgG. For biotinylation, 3 µL of a 0.9 nmol/µL Sulfo-NHS-LC biotin solution (EZ-Link Micro Sulfo-NHS-LC Biotinylation kit, Thermo Scientific, Waltham, MA, USA) was added to approximately 40 µg of IgG (challenge ratio: 10:1) and mixed immediately on a vortex mixer. After 2 h incubation at 23 °C, excess biotin was removed by buffer exchange into MSD conjugate storage buffer (PBS, pH 7.4 containing 0.05% sodium azide) on a Zeba Spin Desalting column (40 K MWCO, 0.5 mL). For storage at −20 °C, glycerole was added to a final concentration of 50% (*v*/*v*). For SULFO-TAG labeling, 1.8 µL of a 3 nmol/µL solution of MSD Gold SULFO-TAG NHS-Ester (Mesoscale Discovery, Rockville, MD, USA) was added dropwise to approximately 40 µg of buffer exchanged IgG (see above, challenge ratio: 20:1). After mixing by pipetting up and down, the reaction was incubated for 2 h at room temperature in the dark. The remaining reagent was removed by buffer exchange into MSD conjugate storage buffer on a Zeba Spin desalting column (40 K MWCO, 0.5 mL). Storage: at 4 °C in the dark.

For the biotinylation of mAb 101-1-1 on a larger scale, 159 µL of 101-1-1 [6.3 mg/mL] was mixed with 841 µL of PBS (0.1 M sodium phosphate/0.15 M NaCl, pH 7.2, Thermo Scientific, Waltham, MA, USA) and buffer exchanged into PBS (0.1 M sodium phosphate/0.15 M NaCl, pH 7.2, Thermo Scientific) on a Zeba Spin desalting column (5 mL, 7000 MWCO, Thermo Scientific, Waltham, MA, USA). Then, 7.4 µL of a 9 nmol/µL Sulfo-NHS-LC biotin stock solution was added (challenge ratio 10:1) and mixed on a vortex immediately. After incubation for 2 h at 23 °C, excess biotin was removed by buffer exchange into PBS. The protein concentration was measured by microplate BCA protein assay (PIERCE/ThermoFisher Scientific, Waltham, MA, USA) and the level of biotin incorporation was determined by HABA (4-hydroxyazobenzene-2-carboxylic acid) assay in a microplate according to the instructions to the EZ-Link Sulfo-NHS-Biotinylation Kit (Themo Scientific, Waltham, MA, USA). BSA and sodium azide were added to the biotinylated antibody to final concentrations of 1% and 0.05%, respectively. For long term storage, half of the material was aliquoted and frozen at −80 °C. The remaining biotinylated antibody stock was supplemented with 40% (*v*/*v*) ethylene glycol and stored in aliquots at −20 °C.

Urea-SDS-PAGE and semi dry blotting: Aβ-peptides were separated by urea-bicine/bis-tris/tris/sulfate SDS-polyacrylamide gel electrophoresis [31] on 12%T/5% C gels and subsequently transferred on PVDF membranes by semi dry blotting, essentially as described previously [32,33]. The protein transfer occurred at a constant current of 0.8 mA/cm^2^ for 30 min with a voltage limit of maximum 30 V.

Bis-Tris Gradient Gel electrophoresis and Western blotting: Proteins and peptides were separated on a 4–12% Bis-Tris gradient gel (Anamed Elektrophorese GmbH, Groß-Bieberau, Germany) at 200 V for 30 min with MES (2-(N-morpholino)ethanesulfonic acid) running buffer (50 mM MES, 50 mM Tris, 0.1% *w*/*v* SDS, 1 mM EDTA). NuPAGE antioxidant (Thermo Fisher Sientific, Waltham, MA, USA) was added to the upper buffer chamber. The proteins were subsequently blotted onto PVDF membranes using 25 mM Bicine, 25 mM Bis-Tris pH 7.2, 1 mM EDTA, and 10% (*v*/*v*) methanol transfer buffer with NuPAGE antioxidant at 20 V for 60 min (MiniGel Tank and Blot Module obtained from Invitrogen/Thermoscientific, Waltham, MA, USA).

Immunohistochemistry: Experiments involving animal tissues were approved by the local animal care and use committee (Landesamt für Verbraucherschutz und Lebensmittelsicherheit (LAVES), Lower Saxony). Brain tissue slides from 12-month-old 5XFAD [34] and 15-month-old APP/^Ld^ transgenic mice [35] were stained as published elsewhere [36]. In brief, 4 µm paraffin sections were deparaffinized in xylene and rehydrated in a series of ethanol, followed by incubation in PBS containing 1% H_2_O_2_ to block endogenous peroxidases. Antigen-retrieval was carried using microwave treatment in 0.01 M citrate buffer pH 6.0 and incubation in 88% formic acid. Non-specific binding sites were blocked with 4% skim milk in PBS containing 10% fetal calf serum. This was followed by overnight incubation with 101-1-1 (1:10,000) or APP C-term (A8717, Sigma Aldrich, Munich, Germany, 1:3000) in a humid chamber. Staining was visualized using the ABC method with a Vectastain Kit (Vector Laboratories, Burlingame, CA, USA) and diaminobenzidine. In the case of fluorescent staining, 101-1-1 (1:6000) and the Aβ_42_-specific antibody D3E10 (1:1000) (Cell Signaling Danvers, MA, USA) were used and detected using anti-mouse-DyLight-594 or anti-rabbit-Dylight-488 antibodies (both Thermo Fisher, Waltham, MA, USA).

Manual and automated immunoprecipitations: Magnetic bead immunoprecipitations (IPs) by hand starting from 400 µL of EDTA-blood plasma were performed as described previously [21]. An adapted protocol for a semi-automated IP procedure was executed on a CyBio FeliX liquid handling Instrument (Analytik Jena, Jena, Germany) equipped with BioShake 3000-Telm (Q Instruments, Jena, Germany) and MAGNUM FLX Enhanced Universal Magnet Plate, Alpaqua Engineering, LLC, Beverly, MA, USA.

Aliquots of EDTA-blood plasma samples (approximately 500 µL per sample), which had been stored at −80 °C in Matrix vials, were thawed and transferred into 1.5 mL reaction vials. The vials were mixed vigorously on a vortex mixer (5 × 10 s), and insoluble material was pelleted by centrifugation for 10 min at 10,000× *g* at room temperature in a fixed angle rotor. Then, 220 µL of each supernatant was transferred into a 96-well sample plate (Deep Well MegaBlock^®^, 96 wells, 2.2 mL, PP (Sarstedt, Nümbrecht, Germany)) and manually mixed with 220 µL of H_2_O.

The sample plate was mounted into the CyBio FeliX instrument and preparation of immunoprecipitation mixes was carried out automatically by adding 100 µL of 5× IP buffer concentrate [21] and 25 µL of 1E8 magnetic beads to 400 µL of the diluted samples. Finally, the instrument transferred the preparations into a process plate (Deep Well MegaBlock®, 96 wells, 2.2 mL, PP (Sarstedt, Nümbrecht, Germany)).

The process plate was then manually placed in the “process” position of the CyBio FeliX instrument and the immunoprecipitation process was executed automatically. The process included an 18-h incubation at room temperature, where the samples were regularly mixed. Then, the process plate was moved into a magnet position for 120 s for immobilization of the magnetic beads. The supernatant was aspirated and discarded, and the process plate was moved back to the process position. Per well, 1 mL of wash buffer (PBS containing 0.1% *w*/*v* BSA) was added and the beads were washed for 5 min at room temperature with agitation. The plate was moved to the magnet position for bead immobilization and removal of the wash fluid as described. Then, the plate was moved back to the process position for a second wash. In total, the magnetic bead immune complexes were washed 3× for 5 min with PBS/0.1% *w*/*v* BSA and once for 3 min with 1 mL of 10 mM Tris-HCl, pH 7.5. After removal of the wash buffer, 2 × 25 µL of elution buffer (20 mM Bicine, pH 7.6, 0.6% *w*/*v* CHAPS) were added, and the beads were resuspended by aspirating and dispensing. The resuspended beads were then transferred into a preheated elution plate (Deep Well MegaBlock®, 96 wells, 1.2 mL PP (Sarstedt, Nümbrecht, Germany)) positioned on the BioShake and incubated for 5 min at 95 °C and 800 rpm to allow elution of the bound Aβ peptides. Then, the suspension was quantitatively moved to a 96-well plate (Deepwell plate 96/500 µL Protein LoBind (Eppendorf, Hamburg, Germany)) located on the magnet and incubated for 120 s for cooling and bead immobilization. Subsequently, 190 µL of Diluent-35 was added to each well (4.8-fold dilution of the IP eluates) and the samples were mixed. Finally, 200 µL of each diluted IP eluate was transferred into a separate plate (Deepwell plate 96/500 µL Protein LoBind (Eppendorf, Hamburg, Germany)). Aliquots of 60 µL each were manually pipetted into 1.5 mL reaction vials and frozen at −80 °C.

Measurements of Aβ_40_ and Aβ_42_: Aβ_40_ and Aβ_42_ levels in 4.8-fold diluted IP eluates were measured with the V-Plex Aβ panel 1 (6E10) multiplex assay kit (Mesoscale Discovery, MSD, Rockville, MD, USA) according to the manufacturer’s instructions and as described previously [21]. In the current version of the two-step immunoassay protocol, the 4.8-fold diluted IP eluates were measured after storage at −80 °C and singular thawing.

Development of the Aβ_−3–40_ immunoassay: The novel sandwich immunoassay for the measurement of Aβ_−3–40_ was developed on the MSD technology platform (Mesoscale Discovery, MSD, Rockville, MD, USA) using MSD Gold 96-well Small Spot streptavidin plates and various MSD reagents, including 20× wash buffer, Diluent-100, Diluent-35, Blocker A, 4× Read buffer, and SULFO-TAG Aβ_40_ detection antibody. In the final assay protocol (SOP), 150 µL of 3% (*w*/*v*) Blocker-A in wash buffer was added to the wells of a 96-well Small Spot streptavidin plate. The plate was sealed with an adhesive foil and incubated for 1 h at room temperature with shaking (500 rpm) on an Eppendorf Thermomix with a lightproof cover. The blocking solution was discarded and the plate was washed three times with 200 µL of wash buffer per well. For plate coating, 25 µL of a 2 µg/mL dilution of the biotinylated capture antibody in Diluent-100 was added per well. The plate was sealed with an adhesive foil and incubated for 1 h at room temperature with shaking (as described above). After three washes with 200 µL of wash buffer per well, 25 µL of sample or calibrator dilution per well was pipetted into the assay plate. The plate was sealed with an adhesive foil and incubated for 1 h at room temperature with shaking (see above). After three washes with 200 µL of wash buffer per well, 25 µL of detection antibody diluted in Diluent-100 was added to each well. Again, the plate was sealed with an adhesive foil and incubated for 1 h at room temperature with shaking (500 rpm) on an Eppendorf Thermomix with a lightproof cover. After three washes with 200 µL of wash buffer per well, 150 µL of 2× Read buffer was added to each well by reverse pipetting. Formation of bubbles must be avoided at this stage. The electro-chemiluminescent signals were recorded on an MSD Quickplex SQ120 reader and analyzed with the MSD Discovery Workbench Software version 4.0.12.

The synthetic calibrator peptide Aβ_−3–40_ was synthesized as described before [25]. The lyophilized peptide was initially solubilized in DMSO (dimethyl sulfoxide) at a concentration of 1 mg/mL and stored in aliquots at −80 °C. Starting from this master stock solution in DMSO, a 600 ng/mL calibrator stock solution for use in the novel Aβ_−3–40_ immunoassay was prepared in Diluent-35 and stored in aliquots for single use at −80 °C. In most experiments, seven serial calibrator dilutions in Diluent-35 plus a zero calibrator (blank) were measured for calculating the standard curve by four-parameter logistic curve fitting. The seven calibrator solutions (standards) were prepared by serial fourfold dilution in Diluent-35 starting with the highest standard to be measured. During the assay validation campaign, in most cases, the highest standard had a concentration of 1875 pg/mL. For the study of the clinical sample, the highest standard contained 7.5 ng/mL of Aβ_−3–40_. In one experiment, an extended fourfold calibrator dilution series including ten fourfold serial dilutions starting at 120 ng/mL plus zero calibrator (blank) was analyzed to determine the ULOQ. The alternative synthetic D7-Calibrator (VKMDAEFRH followed by 5× PEG2 (8-amino-3,6-dioxaoctanoic acid) and the Aβ_40_ C-terminus GLMVGGVV) (Mr: 2571.4)) was synthesized by Biosyntan (Berlin, Germany).

Cell culture: H4 cells stably overexpressing Swedish mutant APP751SWE [37] were cultured in D-MEM/F12, supplemented with 10% fetal calf serum, 2 mM L-Alanyl-L-Glutamine, non-essential amino acids, and 500 µg/mL Hygromycin. To generate H4 cells stably overexpressing wild type APP751, the human amyloid beta A4 protein isoform b precursor cDNA (also known as PreA4 751, APP 751) was subcloned into a pCI-neo mammalian expression vector (Promega, Walldorf, Germany). H4 neuroglioma cells from the exponential growth phase were seeded at 5 × 10^5^ cells/cm² in H4 growth medium (DMEM supplemented with 4.5 g/L glucose, 4 mM L-glutamine, 10% (*v*/*v*) fetal bovine serum, 100 IU/mL penicillin, and 100 μg/mL streptomycin) 24 h before the transfection. Medium changes with serum free DMEM/Ham´s F12 with G5 supplement were performed 3 h before and directly before the transfection. Plasmid DNA and CaCl_2_ were mixed with N,N-Bis(2-hydroxyethyl)-2-aminoethanesulfonic acid (BES) buffer with pH 6.98, yielding final concentrations of 25 mM BES, 140 mM NaCl, 0.75 mM Na_2_HPO_4_, 125 mM CaCl_2_, and 37.5 ng/µL plasmid DNA. For the transfection of a growth area of 9.5 cm², 200 µL of the mixture was incubated for 20 min at room temperature in the dark and subsequently added to the cells. The culture medium was changed to growth medium 24 h after transfection. After 48 h, 500 µg/mL G418 (Thermo Fisher Scientific, Waltham, MA, USA) was added and resistant clones were selected by limiting dilution at <0.2 cells/well in H4 growth medium with 500 µg/mL G418. The cells were maintained in the presence of 500 µg/mL G418. Six clones were isolated and assessed for the level of APP expression. The cells were cultured in DMEM GlutaMAX (Thermo Fisher, Waltham, MA, USA) supplemented with 10% superior fetal bovine serum (Biochrom, Berlin, Germany) and 500 µg/mL G418.

Study approval and study cohort. The study was conducted in accordance with the Declaration of Helsinki. The pseudonomized collection of biological samples and clinical data in a local biobank and their use in biomarker studies was approved by the ethics committee of the University of Göttingen (9/2/16). All subjects or their legal representatives gave their informed consent prior to inclusion. The clinical sample comprising *n* = 23 patients with dementia of the Alzheimer’s type (AD-D) and *n* = 17 patients with dementia due to other reasons (OD) has been described in detail in a previous study [21]. The preparation and storage of blood plasma samples according to standardized protocol were described before (ibid.).

Statistical analysis: For statistical analysis and graphical representation of data, we employed GraphPad Prism 8.4 and IBM SPSS Statistics 26.

Normalization between assay plates. On each assay plate in the core validation campaign, we included the measurement of an aliquot of a quality control (QC) IP eluate in four technical replicates. It served to assess the between-run variance of the Aβ_−3–40_ assay and to allow for testing a method for normalization between assay plates. The QC-IP eluate was prepared by running several IPs in parallel from pooled human blood plasma and combining the obtained IP eluates after 4.8-fold dilution to a single QC-IP eluate. Aliquots for single use were stored frozen at −80 °C until the analysis. For normalization, the average concentration of Aβ_−3–40_ in the QC-IP eluate (mean of four independent assay runs) was calculated as follows:

(1) $$c_{\mathrm{avg}}=\;\overset{n}{\underset{i=1}{\sum }}c_{m}$$

where c_m_ = measured concentration, mean of the replicate measurements on the respective assay plate, and c_avg_ = mean concentration (average of *n* = 4 plates considered).

Subsequently, plate correction factors (k) for each assay plate were calculated as follows:

(2) $$k=\frac{c_{\mathrm{avg}}}{c_{m}}$$

Finally, the normalized Aβ_−3–40_ concentrations (c_norm_) were calculated with the following formula:

(3) $$c_{\mathrm{norm}}=c_{m}*k$$

The normalization by the QC IP eluate was applied to the data obtained with aliquots of a set of three individual control plasma samples and a control plasma pool that were analyzed by the complete two-step immunoassay procedure (i.e., IP followed by Aβ_−3–40_ measurement) in four independent experiments. It should be noted that the described normalization procedure considered only the experimental error contributed by the 96-well plate immunoassay, but not the error contributed by the manual immunoprecipitation.

## Figures and Tables

**Table 1:** Cross reactivity of the Aβ_−3–40_ immunoassay.

Sample	Concentration (pg/mL)	Signal	Calculated Concentration (pg/mL)	% Recognized
Aβ_1–40_	4000	93	below detection range	n.a.
Aβ_1–40_	20,000	79	below detection range	n.a.
Aβ_−__3–38_	4000	93	below detection range	n.a.
Aβ_−__3–38_	20,000	92	below detection range	n.a.
Aβ_−__3–42_	4000	139	12.5	0.31
Aβ−_3–42_	20,000	594	51.6	0.26
Aβ_1–42_	4000	110	below detection range	n.a.
Aβ_1–42_	20,000	85	below detection range	n.a.
sAPPα	320,000	91	below detection range	n.a.
zero calibrator	0	88	n.a.	

n.a.: not applicable, APP: amyloid precursor protein.

**Table 2:** Day-to-day variance of the Aβ_−3–40_ assay.

Assay Run	Mean Signal	Concentration (pg/mL) *
1	6755	193.60
2	7400	195.49
3	7022	204.52
4	7924	279.16
5	8216	214.36
Mean	7463.40	217.43
SD	608.12	35.48
%CV	8.15	16.32

* on each assay plate, an aliquot of the same original diluted quality control (QC) immunoprecipitation (IP) eluate was measured in four technical replicates. The indicated concentrations refer to the mean calculated concentration in the diluted IP eluate without correction for dilution.

**Table 3:** Specificity of the two-step immunoassay.

Sample	Signal *	Concentration (pg/mL) *
Zero calibrator (blank)	94	0
HT7 magnetic bead IP (anti Tau) from plasma	87	Below detection range
1E8 magnetic bead IP from plasma	9140	239.4
1E8 magnetic bead IP from Aβ depleted plasma	150	12.34
1E8 mock IP from PBS/0.1% *w*/*v* BSA	86	Below detection range

* The indicated signals and calculated concentrations are the means of three technical replicates of the control IP eluates and two technical replicates (duplicate reads) of the zero calibrator (blank) on the assay plate. PBS: phoshate buffered saline, BSA: bovine serum albumin.

**Table 4:** Analysis of assay interference.

Spike	Measured Aβ_−3–40_ Concentration(pg/mL) *	Recovery (% of Control)
Control (no spike)	183.93	100
Aβ_1–40_, 4.5 ng/mL	185.72	101
Aβ_1–40_, 13.7 ng/mL	188.39	102
Aβ_1–40_, 45 ng/mL	174.96	95
sAPPα, 40 ng/mL	166.58	91
sAPPα, 120 ng/mL	154.51	84
sAPPα, 363 ng/mL	136.16	74
Aβ_−23–16_, 4.5 ng/mL	162.23	88
Aβ_−23–16_, 13.7 ng/mL	141.70	77
Aβ_−23–16_, 45 ng/mL	88.18	48

* The indicated concentrations are the means of four (control) or two (spiked IP eluates) replicate measurements on the assay plate.

**Table 5:** Efficiency of the 1E8 magnetic bead immunoprecipitation.

Sample	1E8-IP Round 1	1E8-IP Round 2
Signal *	8536	193
Concentration (pg/mL)	229.77	17.29

* Mean of *n* = 3 technical replicates measured on the same assay plate.

**Table 6:** Analytical spike recoveries.

		**Measured Concentration (pg/mL)**			
	**Spike Level**	**Neat**	**Low**	**Medium**	**High**
Sample 1		266.6	334.6	382.8	413.1
Sample 4		243.1	265.0	381.4	468.5
Plasma pool		213.4	281.9	350.9	400.6
Control *		n.a.	n.d.	n.d.	254.7
		**Spike Recovery (pg)**			
	**Spike Level**	**Neat**	**Low**	**Medium**	**High**
Sample 1		n.a.	9.8	16.7	21.1
Sample 4		n.a.	3.2	19.9	32.5
Plasma pool		n.a.	9.9	19.8	27.0
Control *		n.a.	n.a.	n.a.	36.7
		**Spike Recovery (%)**			
	**Spike Level**	**Neat**	**Low**	**Medium**	**High**
Sample 1		n.a.	97.8	83.7	52.7
Sample 4		n.a.	31.6	99.6	81.1
Plasma pool		n.a.	98.6	99.0	67.4
Control *		n.a.	n.a.	n.a.	91.7

* IP from 400 µL of Diluent-35 spiked with 40 pg of Aβ_−3–40_; n.a.: not applicable; n.d.: not determined.

**Table 7:** Repeatability of manual IPs performed in parallel.

	Aβ_−3–40_ pg/mL					
	IP 1	IP 2	IP 3	Mean	SD	%CV
Sample 1	237.45	211.09	233.63	227.39	14.24	6.26
Sample 2	217.73	211.88	217.60	215.74	3.34	1.55
Sample 3	183.42	207.80	203.71	198.31	13.06	6.58

Overall mean coefficient of variation (CV): 4.8%.

**Table 8:** Intermediate imprecision of the two-step immunoassay and normalization *.

	**Measured Concentrations (c_m_) Aβ_−3–40_ (pg/mL)**						
**QC-Sample**	**Plate 1**	**Plate 2**	**Plate 3**	**Plate 4**	**Mean c_m_**	**SD**	**%CV**
QC-1	185.0	150.2	221.0	323.0	219.8	74.6	33.9
QC-2	258.0	160.2	257.3	336.0	252.9	72.0	28.5
QC-3	180.8	145.4	242.0	298.4	216.7	67.6	31.2
QC-pool	227.2	192.7	234.2	336.4	247.6	61.9	25.0
					overall mean %CV		29.6
	**Normalized Concentrations (c_norm_) (pg/mL)**						
**QC-Sample**	**Plate 1**	**Plate 2**	**Plate 3**	**Plate 4**	**Mean c_norm_**	**SD**	**%CV**
QC-1	208.5	167.7	235.7	252.4	216.1	37.0	17.1
QC-2	290.8	178.9	274.5	262.2	251.7	49.9	19.8
QC-3	203.7	162.3	258.2	233.2	214.4	41.2	19.2
QC-pool	256.1	215.0	249.8	246.0	246.0	21.3	8.7
					overall mean %CV		16.2

* Aβ_−3–40_ levels (c_m_) in three individual QC-plasma samples and one QC-plasma pool were determined in four independent runs of the two-step immunoassay (i.e., IP + immmunoassay). Normalized concentrations (c_norm_) were calculated by means of specific correction factors for each assay plate.

**Table 9:** Comparison of means (log2 transformed data) between the diagnostic groups by multiple *t*-tests. AD-D, Alzheimer’s type dementia; OD, other dementias.

	Aβ_−3–40_ *	Aβ_40_ *	Aβ_42_ *	Aβ_42_/Aβ_40_ *	Aβ_42_/Aβ_−3–40_ *
Significant?	No	No	Yes	Yes	Yes
*p*-value	0.65469	0.89737	0.01677	0.00003	0.00228
Mean of OD	6.022	6.684	3.658	−3.025	−2.364
Mean of AD-D	6.061	6.694	3.483	−3.212	−2.577
Difference	−0.0388	−0.0103	0.1756	0.1873	0.2134
SE of difference	0.0860	0.0792	0.0702	0.0392	0.0652
t ratio	0.4508	0.1299	2.502	4.775	3.271
df	38.00	38.00	38.00	38.00	38.00
Adjusted *p*-value **	0.88076	0.89737	0.04947	0.00013	0.00910

* log2-transformed data; ** corrections for multiple comparisons were done using the Holm–Sidak method, with alpha = 0.05 without assuming a consistent SD. Number of t-tests: 5.

## Supplementary Materials

Supplementary materials can be found at https://www.mdpi.com/1422-0067/21/18/6564/s1.

## Abbreviations

AD	Alzheimer’s disease
Aβ	amyloid β
APP	amyloid precursor protein
PET	positron-emission tomography
mAb	monoclonal antibody
BSA	bovine serum albumin
IP	immunoprecipitation
CHAPS	3-(3-cholamidopropyl) dimethylammonio)-1-propanesulfonate
LLOD	lower limit of detection
LLOQ	lower limit of quantification
HABA	4-hydroxyazobenzene-2-carboxylic acid
BCA	bicinchoninic acid
SOP	standard operating procedure
ULOQ	upper limit of quantification
CV	coefficient of variation
PEG2	8-Amino-3,6-dioxaoctanoic acid
QC	quality control
AD-D	dementia of the Alzheimer’s type
OD	other dementias
ROC	receiver operating characteristic
AUC	area under the curve
BES	N,N-Bis(2-hydroxyethyl)-2-aminoethanesulfonic acid
