# Supplementary material for: Development and Technical Validation of an Immunoassay for the Detection of APP669–711 (Aβ−3–40) in Biological Samples
Source: Int J Mol Sci. 2020 Sep 8;21(18):6564. doi: 10.3390/ijms21186564 (PMC7555726; doi:10.3390/ijms21186564)
Supplement: Supplementary file 1 [file ijms-21-06564-s001.pdf]

International Journal of *Molecular Sciences*

MDPI

Supplementary Figure S1. Monoclonal antibody (mAb) 14-2-4 shows excellent selectivity for A$\beta_{-3\text{-}40}$ on Capillary Isoelectric Focusing Immunoassay

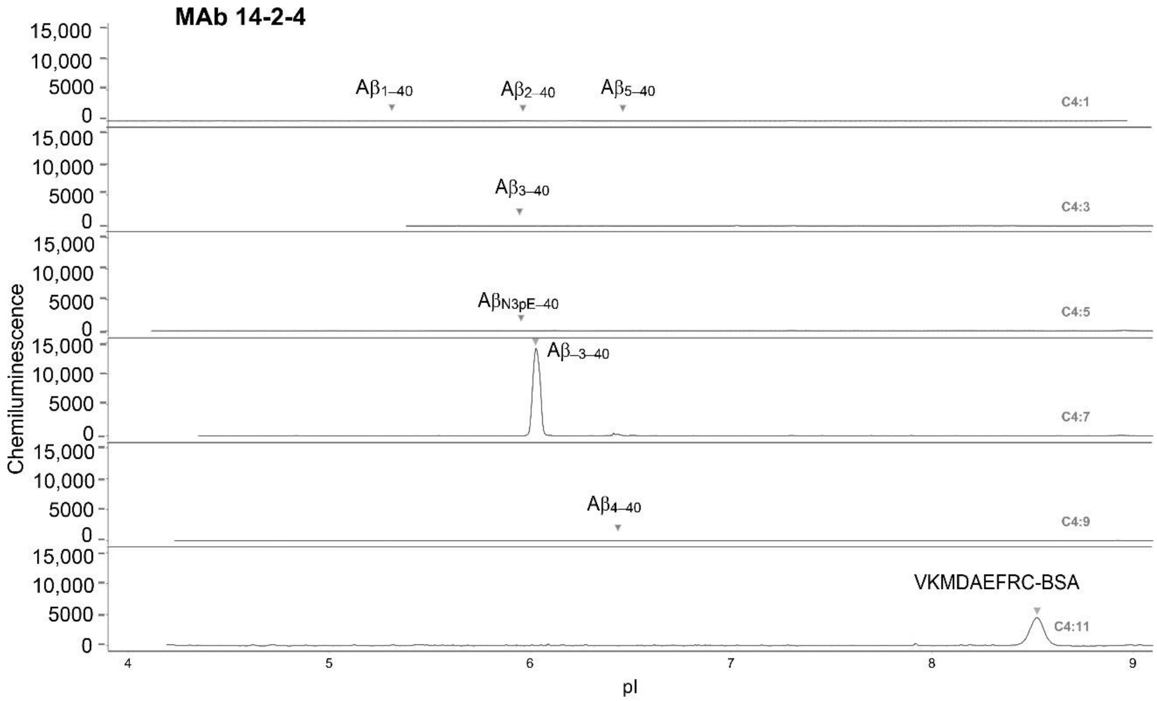

Figure S1. Monoclonal antibody 14-2-4 recognizes A$\beta_{-3\text{–}40}$ with high selectivity on capillary isoelectric focusing immunoassay. A series of synthetic Aβ peptides with different N-termini was separated by isoelectric focusing in microcapillaries, immobilized by a photochemical reaction and probed with mab 14-2-4 (2 µg/mL). Chemiluminescent detection was achieved with biotinylated goat-anti mouse IgG antibody in combination with streptavidin-peroxidase and chemiluminescent substrate. The specific Aβ-variants loaded in the different capillaries as single peptides or in mixtures are indicated. A$\beta_{1\text{–}40}$, A$\beta_{2\text{–}40}$, A$\beta_{3\text{–}40}$, A$\beta_{4\text{–}40}$, A$\beta_{5\text{–}40}$ and A$\beta_{-3\text{–}40}$ were loaded at a concentration of 100 ng/mL. The tested concentration of A$\beta_{N3pE\text{-}40}$ and VKMDAEFRC-BSA was 200 ng/mL.

International Journal of
Molecular Sciences

MDPI

Supplementary Figure S2. MAb 14-2-4 shows high selectivity for $A\beta_{-3-40}$ on Western blot analysis

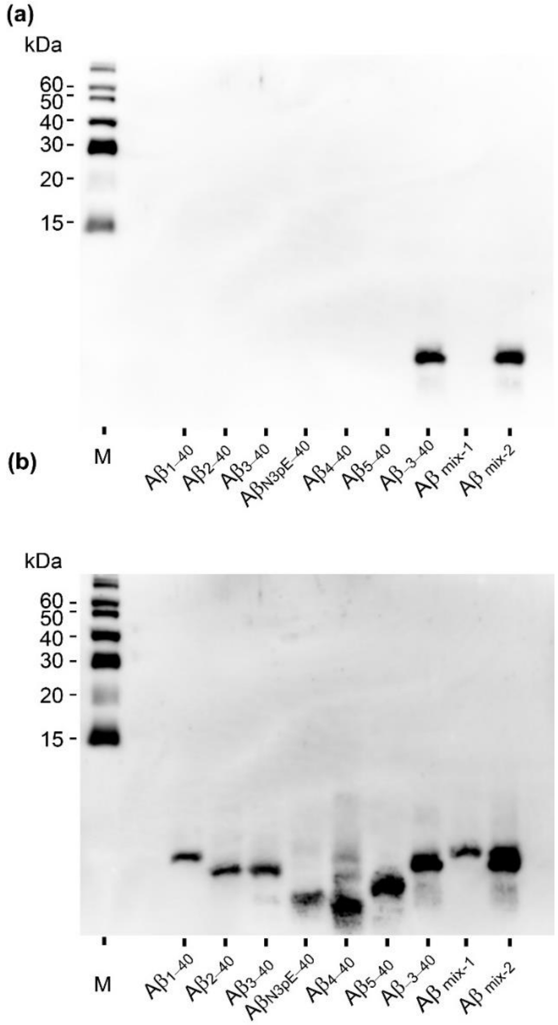

Figure S2. MAb14-2-4 shows high selectivity for $A\beta_{-3-40}$ on Western blot analysis. The indicated synthetic Aβ peptides with different N-termini were separated on a 12%T / 5% C Urea-SDS-polycacrylamide gel, blotted onto PVDF and probed with (**a**) mAb 14-2-4 (1 μg/mL) in combination with goat anti-mouse IgG-peroxidase conjugate (1:30000 dilution in PBS-T). 25 ng of $A\beta_{4-40}$ and $A\beta_{5-40}$ and 10 ng of the remaining peptides were loaded. M) Protein Ladder; Aβ mix-1) mixture containing 10ng, each, of $A\beta_{1-37}$, $A\beta_{1-38}$, $A\beta_{1-39}$, $A\beta_{1-40}$, $A\beta_{1-42}$; Aβ mix-2) mixture containing 10ng each of $A\beta_{1-37}$, $A\beta_{1-38}$, $A\beta_{1-39}$, $A\beta_{1-40}$, $A\beta_{1-42}$ and $A\beta_{-3-40}$. The image was recorded after 5 min exposure. Image display: High: 65535; Low: 0; Gamma: 0.6. (**b**) Reprobing of the same PVDF membrane without stripping with mAb5C3 (anti $A\beta_{40}$) (1 μg/mL) in combination with goat anti-mouse IgG-peroxidase conjugate (1:30000 dilution in PBS-T). Image recorded after 3 min exposure. Image display: High: 65535; Low: 213; Gamma 1.0.

International Journal of
Molecular Sciences

MDPI

Supplementary Figure S3. MAb 101-1-1 immunoprecipitates secreted APPα from conditioned cell culture media

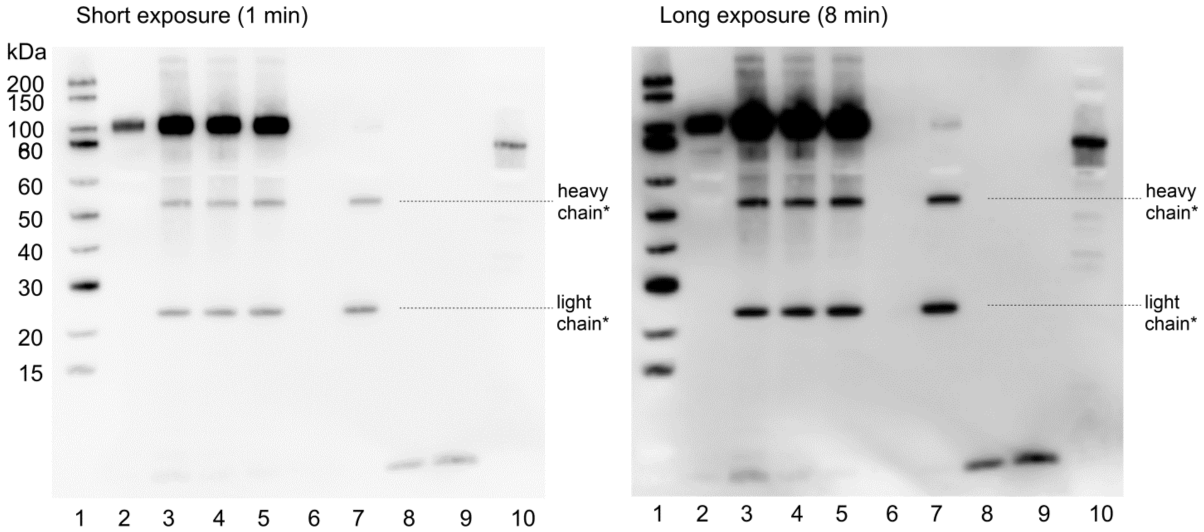

Figure S3. Immunoprecipitation of secreted APP with mAb 101-1-1 from cell culture supernatants. Dynabeads M270 Epoxy were functionalized with mAb 101-1-1 and mixed with aliquots of H4APP751wt cell culture supernatants for overnight immunoprecipitation. The resulting magnetic bead immune complexes were eluted by heating for 10 min at 70°C in LDS-sample buffer (without reducing agent). After addition of 1 mM DTT, the samples were analyzed by SDS-PAGE followed by Western blotting and immunostaining with anti Aβ mAb1E8. Two different exposures (1 min and 8 min) of the same blot membrane are shown. 1) protein ladder; 2) 1 µL of cell culture supernatant H4APP751wt; 3) 101-1-1-M270 magnetic bead IP from 400 µL of conditioned medium; 4) 101-1-1-M270 magnetic bead IP from 200 µL of conditioned medium; 5) 101-1-1-M270 magnetic bead IP from 100 µL of conditioned medium; 6) LDS sample buffer; 7) 101-1-1-M270 magnetic bead control IP from 100 pg/mL of synthetic $A\beta_{3-40}$ in Diluent-35; 8) 100 pg of $A\beta_{3-40}$; 9) 100 pg of $A\beta_{1-40}$; 10) 1 ng of recombinant sAPPα (stock solution prepared in Diluent-35). Image display: Min: 0, Max: 65535, Gamma: 0.65.

*Tentative assignment: Protein bands of approx. 25 and 55 kDa detected in IP-eluates presumably represent heavy and light-chains of mAb 101-1-1 eluted from the magnetic beads.

International Journal of *Molecular Sciences*

MDPI

Supplementary Figure S4. Schematic representation of the assay principle

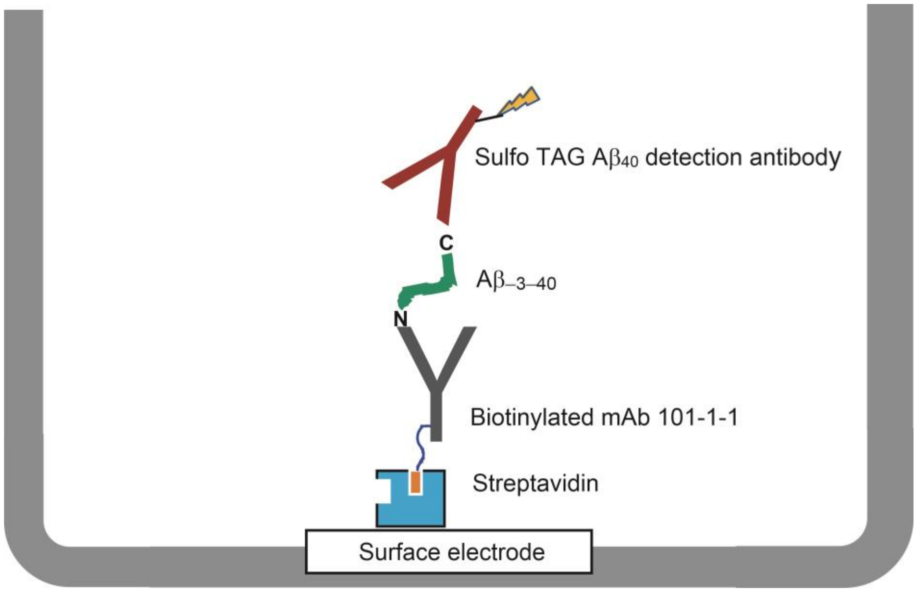

Figure S4. Assay principle. A small spot streptavidin assay plate (MSD) is coated with the biotinylated mAb101-1-1 capture antibody. During the subsequent incubation with the sample, Aβ_{-3-x} is bound to the immobilized mAb101-1-1 via its amino-terminus. For detection, SULFO-TAG labeled anti Aβ_{40} is added, which recognizes selectively the C-terminus of the captured Aβ_{-3-40} peptides. The electro-chemiluminescent signal (light) is generated upon electric stimulation in the presence of read buffer and recorded on the MSD Quickplex SQ120 reader. The graphical representation contains information from the Mesoscale Discovery Web site (https://www.mesoscale.com/en/technical_resources, accessed 2020-07-16. 01:45 p.m.).

International Journal of
Molecular Sciences

MDPI

Supplementary Figure S5. No evidence of systematic positional effects (spatial bias) observed

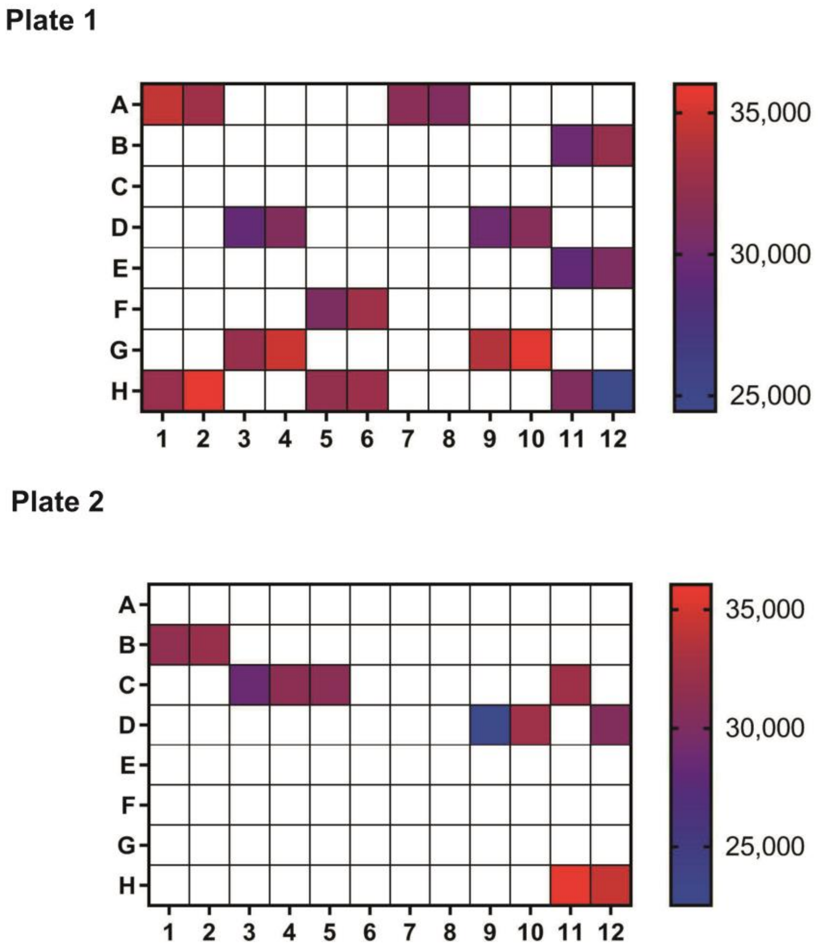

Figure S5. Spatial distribution of signals obtained with technical replicates of a control sample on two different assay plates.

International Journal of *Molecular Sciences*

MDPI

Supplementary Figure S6. Impact of pre-analytical freezing and thawing on the measurements of Aβ38, Aβ$_{40}$, Aβ$_{42}$ and the Aβ$_{42}$/Aβ$_{40}$ ratio

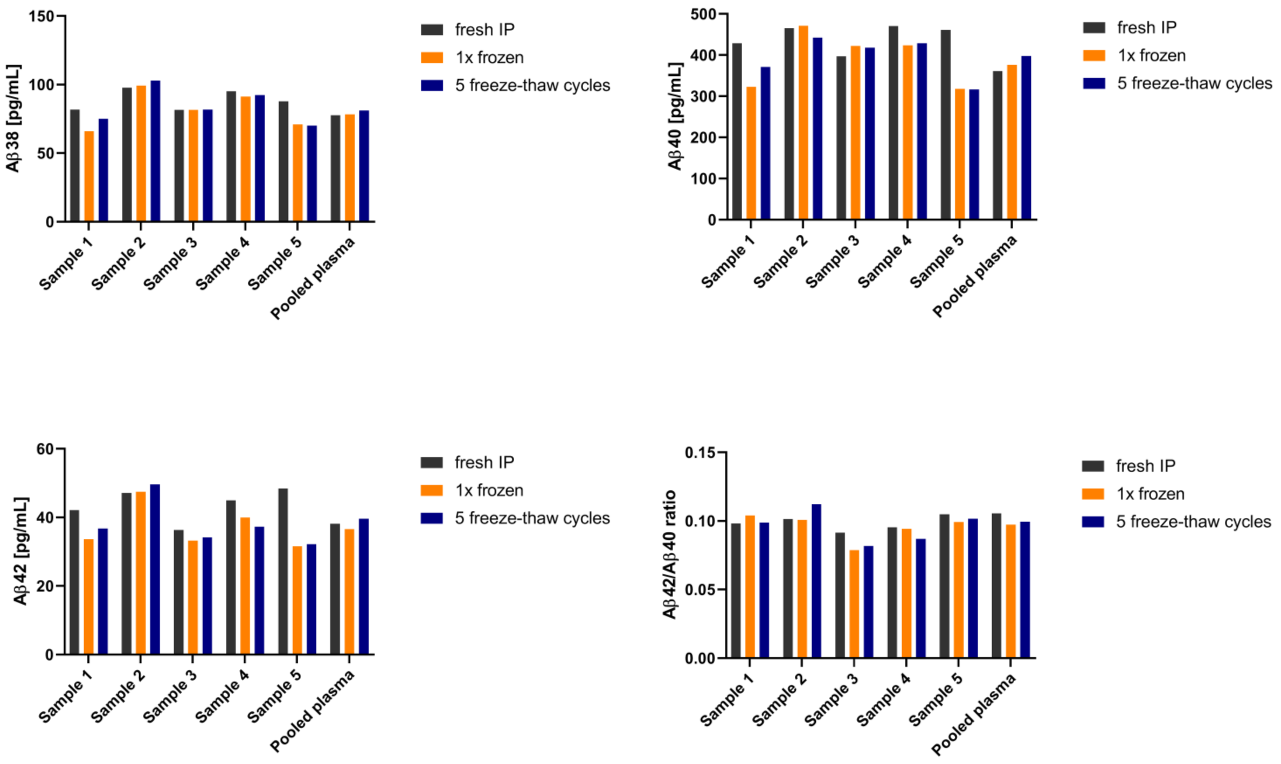

Figure S6. Impact of pre-analytical freezing and thawing on the measurements of Aβ$_{38}$, Aβ$_{40}$, Aβ$_{42}$ and the Aβ$_{42}$/Aβ$_{40}$ ratio. IP-eluates obtained by 1E8 magnetic bead IP from aliquots of 5 individual and 1 pooled plasma sample were diluted with Diluent-35 and measured in the V-Plex Aβ panel 1 (6E10) multiplex assay kit (Mesoscale Discovery, MSD) assay, either freshly or after 1 or 5 freeze-thaw cycles. Mean calculated concentrations from 2 technical replicates on the assay plate are shown. The observations do not suggest a general and systematic influence of single or multiple freeze-thaw cycles on the measurement of the tested Aβ variants or the Aβ$_{42}$/Aβ$_{40}$ ratio in diluted IP-eluates.

International Journal of *Molecular Sciences*

MDPI

Supplementary Table S1. Intra-assay reproducibility of the Aβ-3–40 assay

Table S1. Intra-Assay Reproducibility *.

Sample	Sample 1	Sample 2	Sample 3
mean concentration (pg/mL)	219.93	242.39	224.48
SD	4.78	6.64	4.41
% CV	2.17	2.74	1.96
min	212.31	231.00	218.76
max	224.85	253.14	231.61
n	8	8	8

*Diluted IP eluates obtained from 3 different original EDTA plasma samples were measured on the same assay plate in 8 technical repplicates, each.

Supplementary Table S2. Day-to-day variance of the manual immunoprecipitation procedure

Table S2. Day-to-day variance of the manual magnetic bead IP *.

.	Sample A	Sample B	Sample C	Sample D	Sample E
Day 1	250.13	218.87	219.83	210.36	239.51
Day 2	231.29	193.37	231.34	210.54	266.55
Day 3	196.59	215.44	203.51	197.13	165.53
Day 4	212.30	199.78	216.56	214.06	202.56
mean	222.58	206.87	217.81	208.02	218.54
SD	23.21	12.25	11.45	7.46	44.01
CV (%)	10.43	5.92	5.25	3.59	20.14
Overall mean % CV: 9.1					

* On 4 different days, manual immunoprecipations were executed from aliquots of 5 different original EDTA plasma samples (A–E). IP eluates were diluted 4.8 fold with diluent-35 and stored frozen at −80 °C.After thawing, the Aβ−3–40 concentrations were measured on a single assay plate. Unless otherwise stated, the measured concentrations (pg/mL) are indicated.

International Journal of
Molecular Sciences

MDPI

Supplementary Table S3. Baseline statistics of the measured blood Aβ levels and Aβ ratios in the clinical samp

Table S3. Baseline statistics of the measured Aβ−3–40, Aβ40 and Aβ42 concentrations in the diluted IP-eluates and the concentrations ratios Aβ42/Aβ40 and Aβ42/Aβ−3–40.

	**Aβ−30–40 (pg/mL)**	**Aβ40 (pg/mL)**	**Aβ42 (pg/mL)**	**Aβ42/Aβ40**	**Aβ42/Aβ−30 –40**
n	40	40	404	40	40
min	49.46	73.08	8.65	0.095	0.131
25% precentile	56.74	93.58	10.53	0.105	0.159
Median	65.82	102.2	11.8	0.113	0.174
75% precentile	74.13	113.7	13.42	0.1238	0.201
Max	109.9	155.8	17.05	0.148	0.256
range	60.42	82.76	8.4	0.053	0.125
mean	67.14	104.7	11.92	0.1147	0.1806
SD	13.11	18	1.959	0.012	0.029
SEM	2.073	2.847	0.310	0.002	0.005
p value normality test *	0.00752	0.3287	0.3163	0.1501	0.0672
p value lognormality test *	0.1518	0.9084	0.8412	0.5144	0.4789

* Shapiro-wilk test (alpha = 0.05).

Supplementary Table S4. Shapiro-Wilk tests for normal and lognormal data distribution in the diagnostic groups

Table S4. Shapiro-wilk test for normal and lognormal data distribution in the diagnostic groups.

	***p* value normality test ***	
	OD	**AD-D**
Aβ−3–40	0.0768	0.0331
Aβ40	0.7586	0.5595
Aβ42	0.3655	0.0737
Aβ42/Aβ−3–40	0.9876	0.0257
Aβ42/Aβ40	0.7939	0.832
	***p* value lognormality test ***	
	**OD**	**AD-D**
Aβ−3–40	0.1515	0.5001
Aβ40	0.9445	0.8875
Aβ42	0.3222	0.4832
Aβ42/Aβ−3–40	0.8581	0.8974
Aβ42/Aβ40	0.8925	0.143

* Shapiro-wilk test (alpha = 0.05).
